# Introducing Diradicaloid Thiele's Hydrocarbon Derivatives as Ligand in Transition Metal Chemistry

**DOI:** 10.1002/anie.2880703

**Published:** 2026-08-11

**Authors:** Alok Mahata, Nicolás. I. Neuman, Biprajit Sarkar

**Affiliations:** ^1^ Institut für Anorganische Chemie Universität Stuttgart Stuttgart Germany; ^2^ Institut für Chemie und Biochemie Anorganische Chemie Freie Universität Berlin Berlin Germany; ^3^ Instituto de Desarrollo Tecnológico Para La Industria Química Santa Fe Argentina

**Keywords:** cyclic voltammetry, diradical, electrochromism, metalation, mixed valent, organometallic chemistry

## Abstract

Thiele's hydrocarbon (TH) derivatives, or *p*‐quinodimethanes, are prototypical members of the organic diradicaloid family, predominantly explored in organic and main‐group organometallic chemistry. Herein, we introduce them to transition‐metal coordination by employing N‐doped TH derivatives as ligands. Pyridine‐ and pyrazine‐substituted *p*‐quinodimethane ligands were synthesized, enabling the formation of mono‐ and dinuclear rhodium and cyclometalated iridium complexes (**Py∙Rh**, **Pz∙Rh**, **Py∙Ir**, and **Pz∙Ir**). We show the strong effects that simple metalation, and more importantly, orthometalation can have on the photophysical and electrochemical properties, as well as on singlet‐triplet gaps of these diradicaloids. Additionally, the metalated mixed‐valent forms are strongly electrochromic NIR absorbing dyes. Spectro‐electrochemical studies reveal distinct electronic interactions between the diradicaloid backbone and the metal centers, highlighting the potential of N‐doped TH derivatives as versatile redox‐active ligands in transition‐metal chemistry.

## Graphical abstract

N‐doped Thiele's hydrocarbon derivative is introduced as a ligand for the synthesis of mono‐ and bimetallic rhodium complexes as well as cyclometalated iridium complexes. These compounds constitute the first metal complexes based on a Thiele's hydrocarbon (or *p*‐quinodimethane) derivative and, to the best of our knowledge, the first cyclometalated complexes derived from an organic diradicaloid ligand.

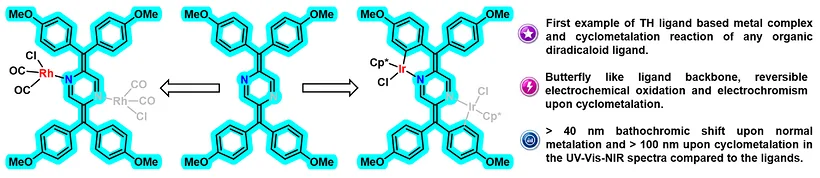

## 1 Introduction

*α*,*α*,*α*′,*α*′‐Tetraphenyl‐*p*‐quinodimethane (**I**), also known as Thiele's hydrocarbon (TH), is considered the most classical organic diradicaloid, first reported by Thiele and coworkers in 1904 [1, 2]. Due to its fascinating photophysical and electrochemical properties and broad range of applications, many organic and organometallic chemists have recently developed various TH derivatives [3, 4, 5, 6, 7, 8, 9]. The electronic ambivalence in these compounds is truly remarkable. TH derivatives or organic diradicaloids in general, can be described by two resonance forms: a closed‐shell quinoid and an open‐shell benzenoid structure (Scheme 1) [10, 11, 12]. Leveraging the unique properties of organic diradicaloids as ligands for transition metals to construct hybrid molecules is one of the most exciting areas in modern chemistry [13, 14, 15]. For example, Anderson and coworkers have reported several tetrathiafulvalene tetrathiolate diradicaloids based Fe, Pt, Pd, and Ni metal complexes (**II**) [16, 17, 18]. Wang group reported an N‐centered diradicaloid where a 1,2‐diketo group in the spacer coordinated to an Ag metal center (**III**) [19]. Recently they extended this idea to a bianthrone system, where they used Al‐ and Si‐based Lewis acids to tune the properties (**IV**) [20].

**SCHEME 1:**
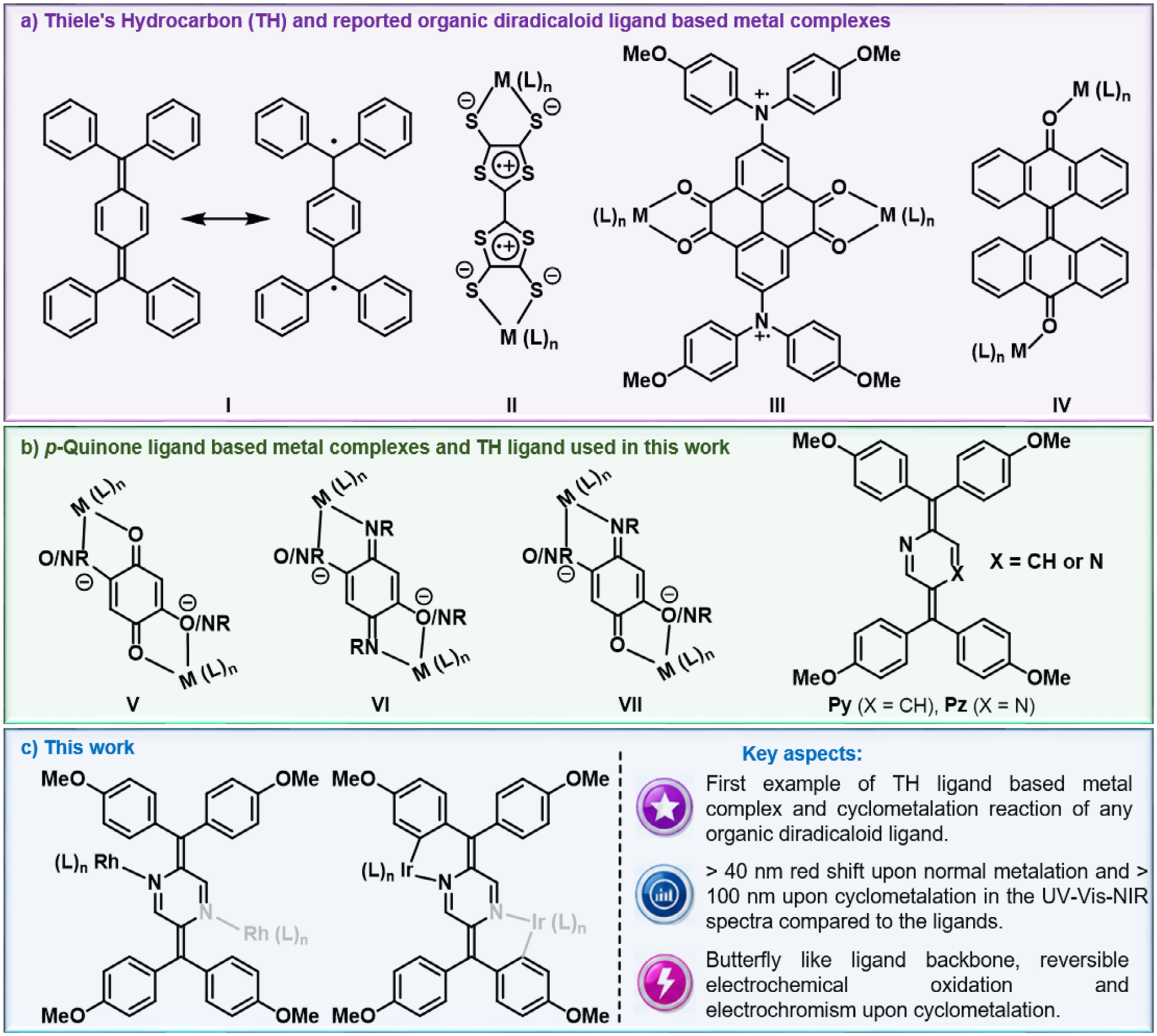
(a–c) Chemical structures of organic diradicaloids as ligand.

Meanwhile, our group, along with many others, has been developing the coordination chemistry of *p*‐benzoquinone (**V**), *p*‐benzoquinone diimine (**VI**), and *p*‐benzoquinone monoimine (**VII**) as redox noninnocent ligands for transition metal complexes over the past decades (Scheme 1) [21, 22, 23, 24, 25, 26, 27, 28, 29, 30, 31, 32, 33, 34, 35, 36, 37]. These complexes are well known for undergoing multiple redox processes, enabling the isolation of mixed‐valent states and single molecular magnets. While the transition metal chemistry of O‐ or N‐based quinone ligands (**V**–**VII**) is well established, no reports exist for their carbon analogue, that is, *p*‐quinodimethane [38, 39, 40], to the best of our knowledge. This absence is likely due to the increased diradical character in *p*‐quinodimethanes [38, 39, 40], which compromises its stability during postmodification [41, 42]. Along with that, the inherently lower donor capability of carbon compared to heteroatoms such as nitrogen or oxygen makes it challenging to design suitable functional groups for effective metal coordination.

Recently, we have reported pyridine and pyrazine spacer‐based TH derivatives or, in other words, N‐doped *p*‐quinodimethanes, that are capable of coordinating to Lewis or Brønsted acids [43, 44]. In this study, we demonstrate their use as ligands in the synthesis of rhodium and cyclometalated iridium complexes. To the best of our knowledge, these represent the first examples of cyclometalated metal complexes synthesized from organic diradicaloid ligands. Additionally, these are also the first examples of transition metal complexes based on TH type organic diradicaloids. However, the introduction of nitrogen heteroatom in the spacer of the TH derivative brings interesting photo‐physical and electronic properties, but it makes the electrochemical oxidation irreversible [43, 44, 45, 46]. Coordination to Lewis or Brønsted acids cannot fix the problem. This study suggests cyclometalation could be an option to resolve this issue.

## 2 Results and Discussion

The starting diradicaloids **Py** and **Pz** were synthesized following our previously reported procedure [44]. Reaction of **Py** and **Pz** with the appropriate equivalent of the (Rh(CO)_2_Cl)_2_ metal precursor in dichloromethane (DCM) at room temperature provides the rhodium complexes **Py∙Rh** and **Pz∙Rh** (Scheme 2). In contrast, synthesis of the cyclometalated iridium complexes required elevated temperatures. **Py∙Ir** and **Pz∙Ir** were obtained by the reaction of the corresponding diradicaloids with [IrCp*(Cl)_2_]_2_ in dichloroethane (DCE) at 70 °C (Scheme 2). All complexes were characterized using ^1^H, ^13^C‐NMR spectroscopy, elemental analysis, and high‐resolution mass spectrometry. ^1^H‐NMR spectra of **Py∙Rh** and **Pz∙Rh** display downfield shift of most of the signals, particularly the N‐C‐H protons compared to starting materials, indicating complex formation (Figures S1–S6). The ^13^C‐NMR spectrum of **Py∙Rh** shows two doublet signals corresponding to two carbonyl groups with a ^1^ *J*_Rh‐C_ coupling constant of around 70 Hz (Figure S2). On the other hand, no significant downfield shift of the N‐C‐H signal was observed in the ^1^H‐NMR spectra of **Py∙Ir** and **Pz∙Ir** complex. Rather the upfield shift of some aromatic signals (between 6.2 and 6.7 ppm) and the disappearance of one aromatic proton from the phenyl ring suggest formation of the cyclometalated complex (Figures S7–S14).

**SCHEME 2:**
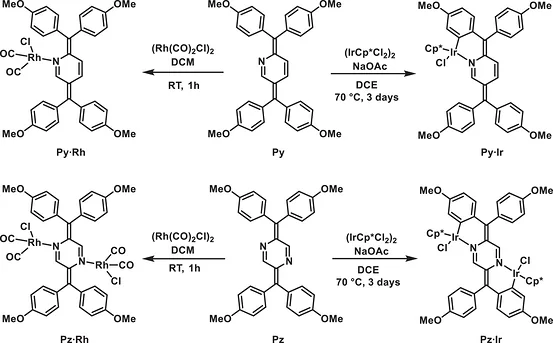
Synthesis of **Py∙Rh**, **Pz∙Rh**, **Py∙Ir**, and **Pz∙Ir**.

Slow diffusion of Et_2_O into concentrated DCM solutions of **Py∙Rh**, **Pz∙Rh**, **Py∙Ir**, and **Pz∙Ir** produced single crystals suitable for single‐crystal XRD measurements [47]. The molecular structures obtained from the crystal data reveal that the C1─C2 bond lengths in **Py∙Rh**, **Pz∙Rh**, **Py∙Ir**, and **Pz∙Ir** are 1.351(3), 1.383(3), 1.373(3), and 1.391(8) Å, respectively, which are comparable to the starting diradicaloid precursors **Py** and **Pz** (Figure 1 and Table 1). Along with that, the C2─C3 bonds exhibit more single bond character (∼1.44 Å) and simultaneously more double bond character is observed for the C3─C4 bond (∼1.33 Å) in case of **Py∙Rh** and **Py∙Ir**, and C3─N1 bond in case of **Pz∙Rh** and **Pz∙Ir** (∼1.31 Å). These data suggest quinoid nature of the ligand backbone in the solid state for the metal complexes, and is compatible to data obtained for the metal‐free diradicaloid ligands. Interestingly, while the central pyridine or pyrazine cores remained nearly planar in the free ligands and in **Py∙Rh** or **Pz∙Rh**, **Py∙Ir** exhibited deviation from planarity to adopt a three‐legged piano‐stool geometry. This distortion is even more pronounced in **Pz∙Ir**, which formed a binuclear complex with a butterfly‐like geometry of the central pyrazine spacer (Figures 1, S19, and S20). Such structural motifs are well‐known in polycyclic aromatic hydrocarbon (PAH) chemistry, particularly in anthraquinodimethane‐based diradicaloids, where similar distortions arise due to steric repulsion between anthracene protons and terminal aryl groups [48, 49]. Recently, Farinola and co‐workers reported the first example of folded TH derivatives with distorted central ring, where the distortion arises from the halogen substituents in the spacer and terminal rings [50]. We show here an intriguing example where comparable distortions and a similar structural motif is induced through cyclometalation driven metal chelation in simple TH derivatives.

**FIGURE 1:**
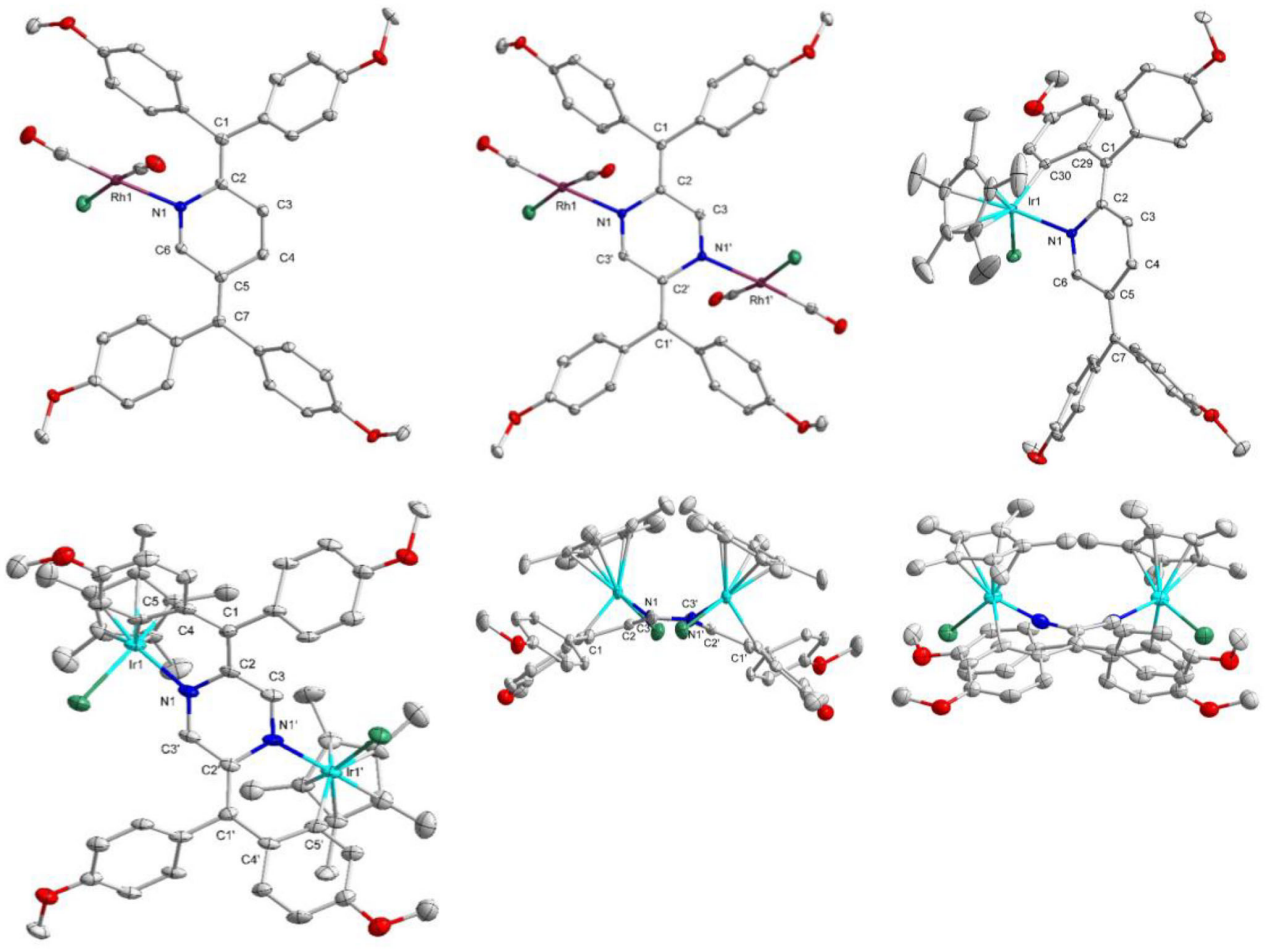
Molecular structure of **Py∙Rh** (top left), **Pz∙Rh** (top middle), **Py∙Ir** (top right), and **Pz∙Ir** (molecule **A**) (bottom left) with thermal ellipsoids at the 50% probability level. All H atoms are omitted for clarity reasons. Molecular structure of **Pz∙Ir** (molecule **A**) from different angles (bottom middle and bottom right).

**TABLE 1:** Selected bond lengths (Å).

Bond	Py∙Rh	Pz∙Rh	Py∙Ir	Pz∙Ir (A)	Pz [44]	TH [2]
C1─C2	1.351(3)	1.384(3)	1.373(3)	1.391(8)	1.376(4)	1.381(3)
C2─C3	1.469(3)	1.434(3)	1.447(3)	1.412(9)	1.435(4)	1.449(3)
C3─C4	1.315(3)	—	1.336(3)	—	—	1.346(3)
C2─N1	1.397(2)	1.422(3)	1.415(3)	1.412(8)	1.416(4)	—
C3─N1	—	1.305(3)	—	1.313(7)	1.300(4)	—
C5─C7	1.358(3)	—	1.382(3)	—	—	—
M─N1	2.1533(15)	2.1174(18)	2.083(2)	2.092(6)	—	—

Ultraviolet–visible–near‐infrared (UV–vis–NIR) spectroscopic measurements of the rhodium complexes (**Py∙Rh** and **Pz∙Rh**) show significant red shift in the absorption maximum (*λ*_max_ = 519 and 522 nm, respectively) compared to the corresponding ligand precursors **Py** and **Pz** (469 and 475 nm, respectively) (Figures S24–S34). The shift is even more pronounced in case of iridium complexes. **Py∙Ir** exhibits an absorption maximum at 524 nm along with a shoulder at lower energy region (574 nm). Furthermore, **Pz∙Ir** exhibits a peak at 622 nm along with a shoulder peak at higher energy region (538 nm) (Figure 2). Time‐dependent density functional theory (TD‐DFT) calculations on the optimized geometry, performed using ORCA 5.0 and 6.0 [51, 52], suggest that the lowest‐energy HOMO to LUMO transitions are predominantly ligand based π → π* transitions (Tables S5–S8). In the case of **Py∙Rh** and **Pz∙Rh**, both frontier molecular orbitals are mostly localized on the central spacer unit, with only minor contributions from the terminal aryl groups (Figures S49 and S51). Whereas in the case of **Py∙Ir** and **Pz∙Ir**, the HOMO contains slight contribution from the metal center as well (Figures S53 and S55). These data show that the frontier orbitals and hence the absorption properties of these TH analogues can be strongly tuned through metalation, and particularly through cyclometalation. Infra‐red (IR) spectra of **Py∙Rh** and **Pz∙Rh** show lower energy shifts of the symmetric and asymmetric stretching frequencies of the CO units compared to the [Rh(CO)_2_Cl]_2_ precursor. Slightly higher frequencies (2006 and 2079 cm^−1^) in case of **Pz∙Rh** compared to **Py∙Rh** (2003 and 2076 cm^−1^), indicate higher π‐accepting ability of pyrazine over pyridine (Figures 2 and S21–S23).

**FIGURE 2:**
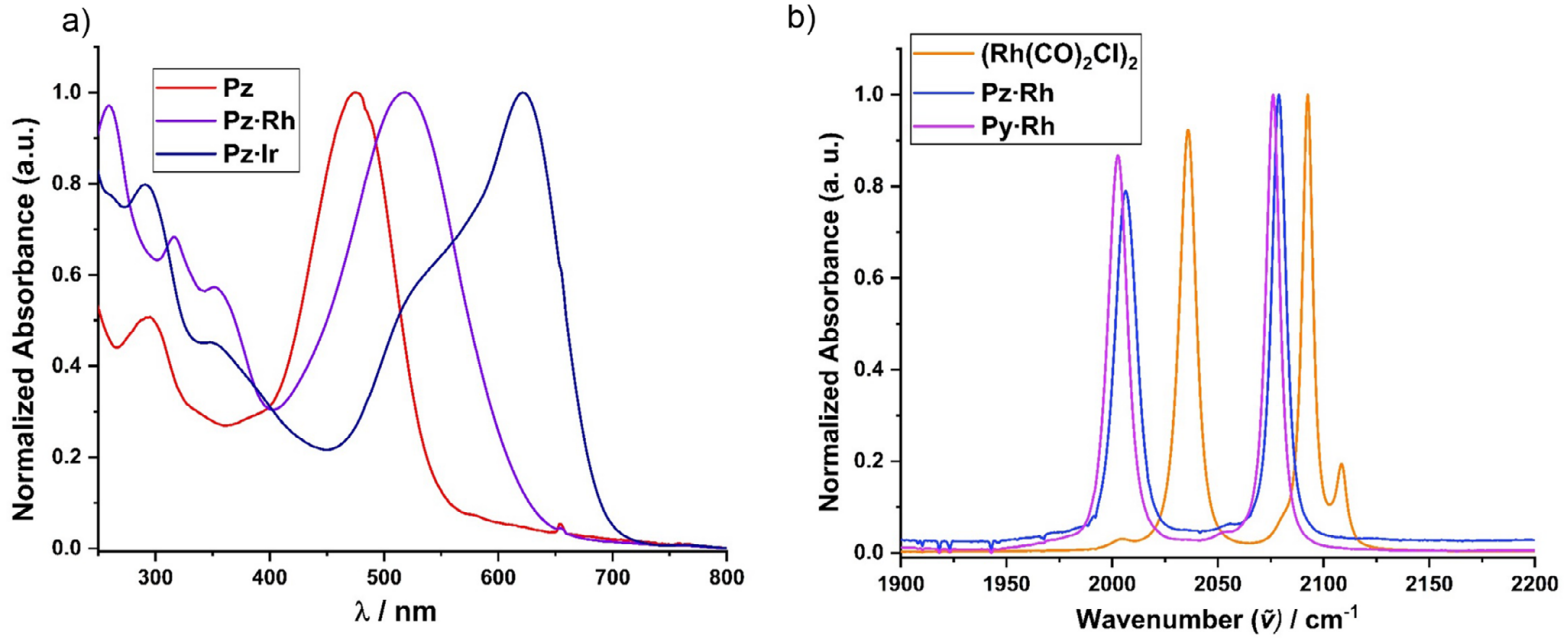
(a) Normalized UV–vis–NIR spectra of **Pz**, **Pz∙Rh**, and **Pz∙Ir** in DCM. (b) Comparison of CO stretching bands in the IR spectra of **Py∙Rh**, **Pz∙Rh** and (Rh(CO)_2_Cl)_2_ in DCM.

Electrochemical oxidations of pyridine or pyrazine spacer‐based TH derivatives reported in the literature are irreversible in nature, possibly because of higher electronegativity of N‐atom compared to carbon [43, 44, 45, 46]. Obtained radical‐cation or di‐cation upon one or two electron oxidations probably undergoes further reactivities through N center. However, the cyclic voltammetry (CV) studies of **Py∙Rh** and **Pz∙Rh** in DCM with Bu_4_NPF_6_ as supporting electrolyte, suggest an expected anodic shift in the oxidation potential compared to **Py** and **Pz**, but the oxidation processes remain irreversible, as was previously observed in the case of the free ligands (Figure 3) [44]. This indicates breaking of Rh─N bond upon oxidation of **Py∙Rh** and **Pz∙Rh** under CV condition.

**FIGURE 3:**
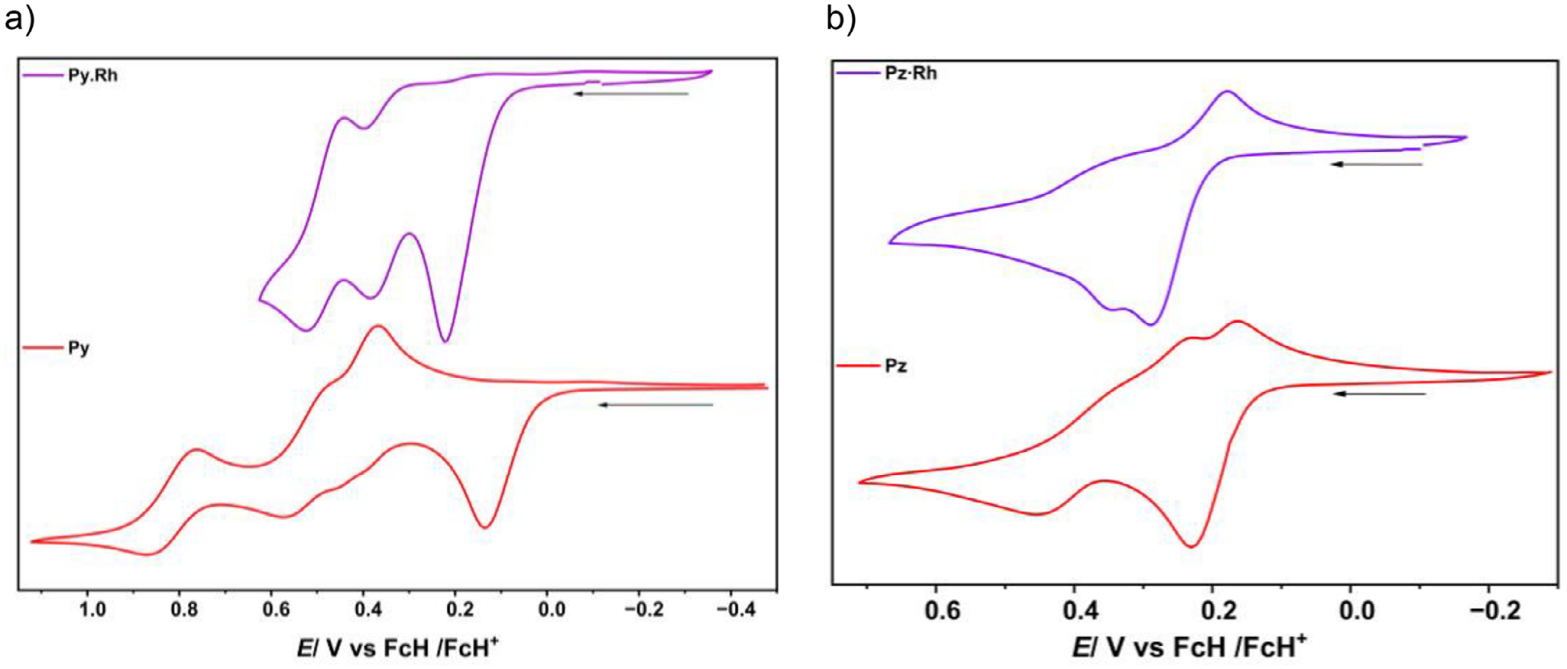
(a) Overlay of the cyclic voltammograms of **Py** and **Py∙Rh** (a), and **Pz** and **Pz∙Rh** (b) measured in DCM/0.1 M Bu_4_NPF_6_ at a GC working electrode at 100 mV s^−1^.

However, in the case of **Py∙Ir** and **Pz∙Ir** two completely reversible one‐electron oxidation processes were seen. In the case of **Py∙Ir** the separation between the two processes (Δ*E*) is very small (Δ*E* = 0.07 V and comproportionation constant, *K*_c_ = 1.5 × 10^1^). A further reversible oxidation step at higher potential (*E*_1/2_ = 0.99 V) was also detected and this process is likely related to the oxidation of the formal Ir(III) center to Ir(IV) (Figures 4 and S38). Interestingly, for the doubly cyclometalated complex **Pz∙Ir**, a separation of 0.169 V (*K*c = 7.2 × 10^2^) between the two redox events was observed, with *E*_1/2_ = 0.094 and 0.263 V versus FcH/FcH^+^ (Figures 4 and S39). This separation increases to 0.28 V, (*K*_c_ = 5.4 × 10^4^) if Bu_4_NBArF_24_ is used as an electrolyte (Figure S41). These observations show that electrostatic interactions do play a substantial role in the observed separation between the redox processes [53, 54]. From the above discussion it is seen that coordination of metal centers, and in particular the cyclometalation step, have a drastic influence on the redox properties of TH derivatives. Not only do the aforementioned perturbations strongly influence the reversibility of the redox steps, they also contribute to a higher thermodynamic stability of the one‐electron oxidized species, as observed by the substantially higher *K*_c_ value for **Pz∙Ir** in comparison to **Py∙Ir**. To the best of our knowledge, this is the first time that such an influence of cyclometalation on redox properties has been investigated for TH derivatives.

**FIGURE 4:**
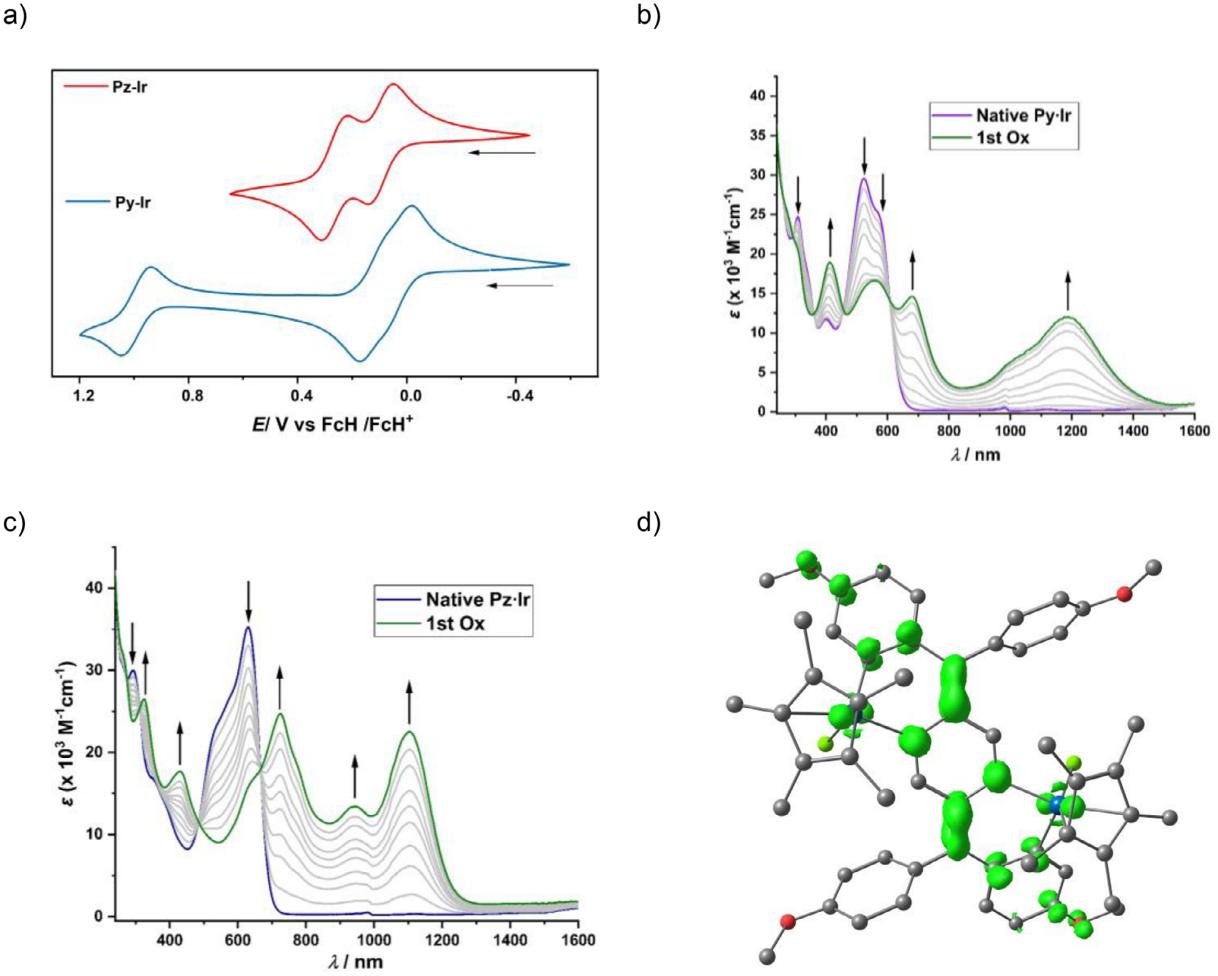
(a) Overlay of the cyclic voltammograms of **Py∙Ir** and **Pz∙Ir** measured in DCM/0.1 M Bu_4_NPF_6_ at a GC working electrode at 100 mV s^−1^. (b) Changes in the UV–vis–NIR spectra of **Py∙Ir** in DCM/0.2 M Bu_4_NPF_6_ during first oxidation with an Au working electrode. (c) Changes in the UV–vis–NIR spectra of **Pz∙Ir** in DCM/0.2 M Bu_4_NPF_6_ during first oxidation with an Au working electrode. (d) Spin density plot of the oxidized species of **Pz∙Ir** (isosurface 0.005).

To further investigate the nature of the oxidized species, UV–vis–NIR spectroelectrochemistry was performed, suggesting the formation of a radical cationic species having absorption (*λ*_max_ = 1105 and 942 nm, in case of **Pz∙Ir**) in the NIR region (Figure 4). DFT calculations suggest that the SOMO of the oxidized species is delocalized over the cyclometalated aryl ring through the pyrazine spacer along with significant contribution from metal d‐orbital (Figure S59). Spin density calculations also support this observation (Figures 4 and S60). Despite the nonplanar, butterfly‐like structure of the spacer, cyclometalation aligns the π‐orbitals of the cyclometalated aromatic ring parallel to the pyrazine ring, enabling effective delocalization. This delocalization stabilizes the radical cation state against disproportionation reactions significantly more than in our previously reported phenyl‐spacer‐based radical cation [44].

The nature of the mixed valence radical cation state according to Robin–Day classification was estimated from the inter valence charge transfer (IVCT) band [55]. The electronic coupling integral (V) was calculated following deconvolution (Figure S48) of the IVCT band using the Markus‐Hush theory, considering the absorption maximum (λ_max_ = 1106 nm, ${\tilde{\nu }}_{\max}$ = 9039 cm^−1^), the bandwidth at half‐height (${\tilde{\nu }}_{1/2}$ = 1353 cm^−1^), the molar absorptivity (*ε* = 22500 M^−1^ cm^−1^), and the distance (*R*) between two radical centers (5.58 Å; from the DFT optimized geometry, as the radical cation is mostly ligand based, two radical carbon atoms considered as redox center) as per Equation 1 [56].

(1) $$V=\{\left[2.06\times 10^{-2}{\left({\tilde{\nu }}_{\max}{\tilde{\nu }}_{1/2}\varepsilon \right)}^{1/2}\right]/R\}$$

Applying Equation 1 to the radical cation of **Pz∙Ir** yields a coupling integral of *V* = 1937 cm^−1^, which is relatively higher than the ground state Creutz‐Taube ion [57, 58], suggesting more delocalization in this case. However, the coupling integral value is less than λ/2 (4520 cm^−1^), recommending its assignment as a borderline class II–class III system [59, 60].

Similarly, in the case of **Py∙Ir** also, after first oxidation formation of an absorption band at 1185 nm was observed. However, the intensity of this low energy band is substantially lower compared to that observed for the binuclear doubly cyclometalated species (Figure 4). Lower stability of the corresponding radical cationic state toward disproportionation further points toward nonplanarity, arising from steric repulsion of aryl terminal group at the C7 radical center, analogous to that observed in the phenyl system [44]. Absorptions beyond 1000 nm (with high extinction coefficients) is only observed for the one‐electron oxidized radical forms of **Py∙Ir** and **Pz∙Ir** (Figure 4), making these complexes intriguing examples of electrochromic NIR dyes. Although there are many reports in the literature on cyclometalated Ir(III) complexes with fascinating photophysical properties and their applications as OLED materials and in photodynamic therapy [61, 62], isolation of oxidized form of such complexes remain challenging, most of the cases even the electrochemical oxidations processes are irreversible [63]. Chemical isolation of the oxidized form of **Py∙Ir** and **Pz∙Ir** remains unsuccessful because of their transient nature, probably it requires different co‐ligands other than Cp* and/or Cl, investigations along this direction currently ongoing in our laboratory.

The relative energies of spin states, natural orbitals, and diradical character [64] of the neutral complexes were calculated with ORCA, using the PBE0 functional with def2‐TZVP basis sets on all atoms, and the resolution‐of‐the‐identity and chain‐of‐spheres approximations [65, 66]. Figure 5 shows the energy differences between the complexes with geometries optimized as closed‐shell singlets (CSS) and triplets (T). In all cases the CSS configuration is lower in energy by 26–39 kJ/mol, displaying shorter C─C quinoid bonds in the range of 1.37–1.38 Å, while the T configurations display longer C─C bonds around 1.43–1.46 Å.

**FIGURE 5:**
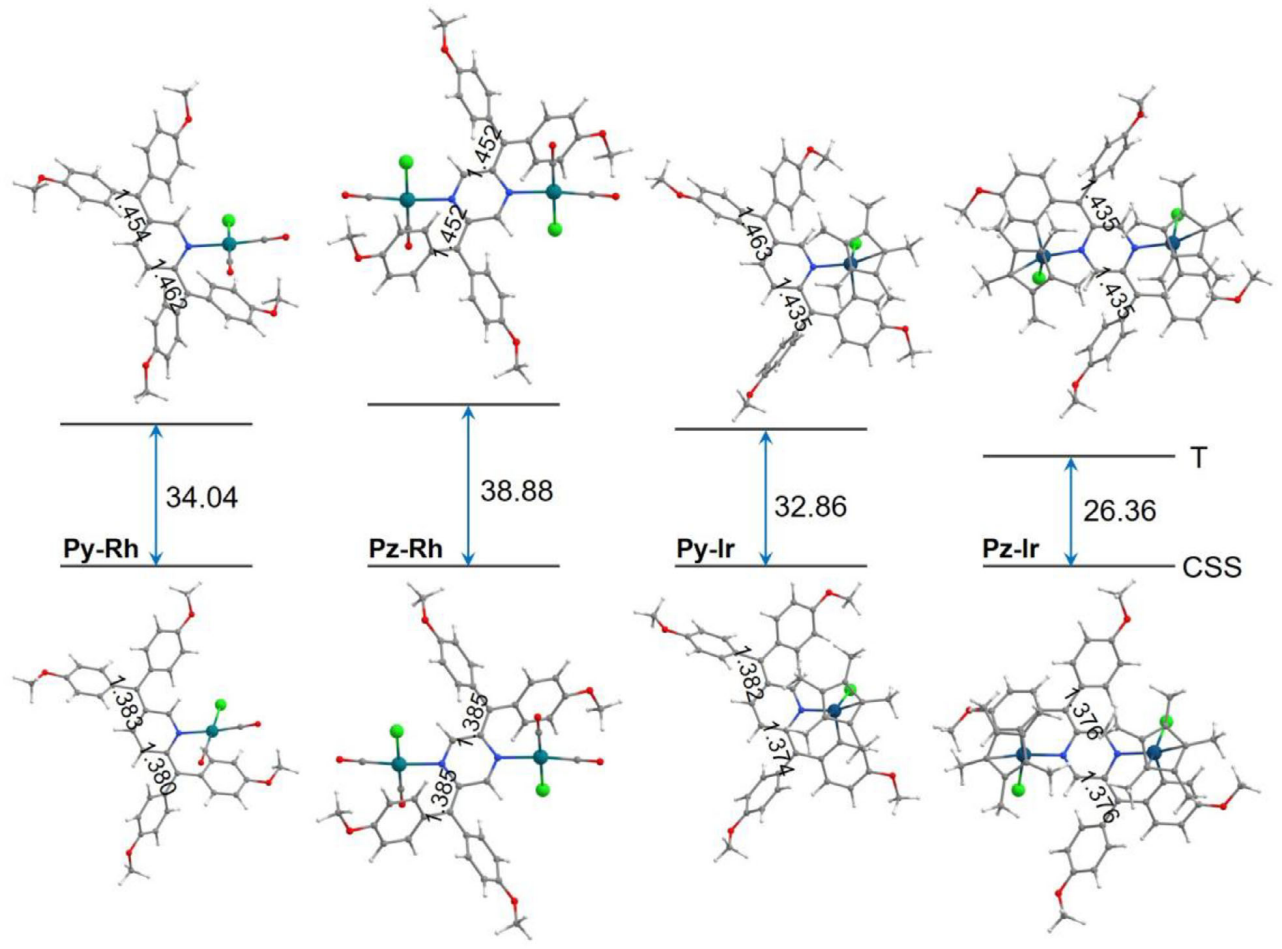
Energy differences (in kJ/mol) between the complexes optimized as closed‐shell singlets (CSS) or triplet (T) states. The quinoid C─C bond lengths for each structure are included.

These results agree with our previous studies on the free‐ligands **Py** and **Pz** (**2^Py^** and **2^Pz^** in the previous paper), reported by us recently [44] with the S‐T gaps of the complexes being lower than those of the ligands (∼56 kJ/mol for **Py** and ∼68 kJ/mol for **Pz**). The highest occupied and lowest unoccupied natural orbitals (HONO and LUNO) for the CSS and T geometries are shown in Figure S62. For the T structures with longer quinoid C─C bond distances, both the HONO and LUNO are centered in the Py or Pz rings, with appreciable delocalization toward the methoxyphenyl substituents. The HONO resembles a quinoid configuration, while the LUNO has a larger character on the methine C, indicating a certain diradicaloid character. This is in line with the calculated diradical character (*y*_0_) values shown in Table S9. The complexes in the T optimized geometry present strong antiferromagnetic couplings in the −900 cm^−1^ to −2400 cm^−1^ range. This result, together with the calculated diradical character value, which is only appreciable for **Py∙Rh** (*y*_0_ = 0.198), indicates that the diradicaloid character of these complexes is low. For the CSS optimized geometries of **Py∙Rh** and **Py∙Ir** the HONO and LUNO are also localized in the central Py ring, while for **Pz∙Rh** and **Pz∙Ir** the HONO and LUNO are centered on the methoxyphenyl substituents. The appearance of the orbitals, the very large antiferromagnetic exchange coupling constants and the *y_0_* values of zero indicate that the ground states of these complexes are closed‐shell.

## 3 Conclusion

In conclusion, we have successfully synthesized rhodium and cyclometalated iridium complexes **Py∙Rh**, **Pz∙Rh**, **Py∙Ir**, and **Pz∙Ir**, using TH derivatives or N‐doped *p*‐quinodimethanes as ligand. To the best of our knowledge, **Py∙Ir** and **Pz∙Ir** represent the first examples of cyclometalated metal complexes derived from organic diradicaloid ligands, as well as the first transition metal complexes with TH type diradicaloids. A bathochromic shift of more than 40 nm in the UV‐vis‐NIR spectra upon metalation could be observed, which becomes much more pronounced (>100 nm) in case of cyclometalated Ir complexes (**Py∙Ir** and **Pz∙Ir)**. Cyclometalation also distorted the planarity of the central ring thus making **Pz‐Ir** a rare example of a compound containing a butterfly like TH backbone. This study opens up the possibility of using redox‐active and optically‐rich TH derivative as ligand system for cyclometalation reaction with optoelectronically versatile 5d metal center, paving the way toward the design of novel hybrid materials. Our findings also demonstrate that the electrochemical behavior (redox potential, reversibility, and stability of the one‐electron oxidized forms) of diradicaloids and their singlet‐triplet gaps can be tuned through coordination to transition metals. Increase in stability of the intermediate radical cation state in case of **Pz∙Ir** suggests possibility of isolating the mixed valence compounds with the C‐analogue of *p*‐Benzoquinone, that is *p*‐quinodimethanes also. These results thus open up a novel way of strongly tuning the physical properties of organic diradicaloids. Apart from the diradicaloid field, our investigations show the possibility of introducing diradical bridges as redox‐active ligands in metal complexes. This aspect might allow manipulation of both physical and chemical properties of such complexes through “spin chemistry.” Further investigations in these directions are currently ongoing in our laboratory.

## Author Contributions

**Alok Mahata**: conceptualization, investigation, writing – original draft, methodology, validation, visualization, formal analysis, data curation. **Nicolás. I. Neuman**: writing – original draft, methodology, visualization, software, formal analysis, data curation, resources. **Biprajit Sarkar**: conceptualization, funding acquisition, writing – review and editing, project administration, supervision, resources.

## Conflicts of Interest

The authors declare no conflicts of interest.

## Supporting information




**Supporting File**: anie73985‐sup‐0001‐SuppMat.docx.

## Data Availability

The data that support the findings of this study are available in the Supporting Information of this article.
