# Supplementary material for: Introducing Diradicaloid Thiele's Hydrocarbon Derivatives as Ligand in Transition Metal Chemistry
Source: Angew Chem Int Ed Engl. 2026 Aug 11;65(41):e2880703. doi: 10.1002/anie.2880703 (PMC13638131; doi:10.1002/anie.2880703)
Supplement: Supplementary file 1 [file ANIE-65-e2880703-s001.docx]

Supporting Information

Introducing Diradicaloid Thiele’s Hydrocarbon Derivatives as Ligand in Transition Metal Chemistry

Alok Mahata, Nicolás. I. Neuman and Biprajit Sarkar*

1. Instrumental ................................................................................................................................. 2

2. Synthetic procedures ................................................................................................................... 3

3. NMR Spectroscopy................................................................................……………………………7

4. Crystal structures and crystallographic data.................................................................................14

5. IR Spectroscopy………………………………………………………………….………………………19

6. UV-vis-NIR Spectroscopy…………………………………………………………………………...…..20

7. Cyclic voltammetry and UV-vis-NIR Spectro-electrochemistry.....................................................26

8. Computational details ...................................................................................................................33

9. References ...................................................................................................................................60

**1. Instrumental**

Unless otherwise noted, all reactions were performed using standard Schlenk-line techniques under an inert atmosphere of argon (Linde, Argon 4.8, purity ≥ 99.998) or an MBraun glove box fitted with a gas purification and recirculation unit. Commercially available chemicals were used without further purification. **Py**, **Pz**, (IrCp*Cl_2_)_2_, NBu_4_BArF_24_ were synthesized using literature reported procedures.^[1]^ Dichloroethane (DCE) was dried and stored over 4Å molecular sieve. Other solvents were available from MBRAUN MB-SPS-800 solvent system and additionally degassed using standard techniques. ^1^H NMR and ^13^C NMR were recorded on Brucker 250 and 500 MHz spectrometer. Chemical shifts are reported in ppm (relative to the TMS signal) with reference to the residual solvent peaks.^[2]^ Multiplets are reported as follows: singlet (s), duplet (d), triplet (t), quartet (q), septet (sept), and multiplate (m). NMRs under the exclusion of air were conducted in J. Young’s NMR tubes oven dried and flushed with argon prior to use.

Cyclic voltammograms were recorded with a Palm Sens 4 potentiostat by working in anhydrous and degassed Dichloromethane with 0.1 м NBu_4_PF_6_ (dried, > 99.0%, electrochemical grade, Fluka) or NBu_4_BArF_24_ as supporting electrolyte. Concentrations of the compounds were about 1·10^-4^ м. A three-electrode setup was used with a glassy carbon working electrode, a coiled platinum wire as counter electrode, and a coiled silver wire as a pseudo-reference electrode. The ferrocene/ferrocenium couple was used as internal reference. UV-vis-NIR spectra were recorded on J&M TIDAS UV-vis-NIR spectrophotometer. UV-vis-NIR spectroelectrochemical measurements were carried out in an optically transparent thin-layer electrochemical (OTTLE)^3^ cell (CaF_2_ windows) with a gold mesh working electrode, a platinum-mesh counter electrode, and a silver-foil pseudo reference. IR spectra were recorded on SHIMADZU IRAffinity-1S FTIR spectrophotometer.

X-ray data were collected on a Bruker Kappa Apex Duo system, Eulerian 4-circle diffractometer or Rigaku XtaLAB Synergy R, DW system four-circle diffractometer at 150(1) K or 100(1) K, using graphite-monochromated MoΚα radiation (λα= 0.71073 Å). The strategy for the data collection was evaluated by using the Smart software. The data were collected by the standard omega scan or omega + phi scan techniques, and were scaled and reduced using Saint+ and SADABS software. The structures were solved by direct methods using SHELXS-97 or intrinsic phasing using SHELXL-2014/7 and refined by full matrix least-squares, refining on F2. Non-hydrogen atoms were refined anisotropically.^[4-6]^ Structures were solved using SHELXS-97 and the software OLEX2, while refinement was carried out on F2 against all independent reflections by the full matrix least-squares method using the SHELXL-97 program. All non-hydrogen atoms were refined using anisotropic thermal parameters.

**2. Synthetic procedures**

Synthesis of **Py∙Rh**

A mixture of **Py** (106 mg, 0.2 mmol, 1 equiv.) and di-μ-chloro-tetracarbonyldirhodium(I) (39 mg, 0.1 mmol, 0.5 equiv.) was added to an oven-dried Schlenk flask and dissolved in dry DCM (20 mL). The reaction mixture was stirred at room temperature for one hour. During this time, the color of the mixture changed from orange-red to pink. All the volatiles were removed under vacuum. 5 mL of diethyl ether (Et_2_O) was added to the residue and obtained solid was collected by filtration, washed with 5 mL of Et_2_O, yielding the compound **Py∙Rh**. Single crystals suitable for XRD were obtained by diffusion of Et_2_O into concentration DCM solution of **Py∙Rh**. Yield: 124 mg (0.17 mmol, 85%).

**^1^H-NMR** (CD_2_Cl_2_, 250 MHz) *δ*: 8.75 (s, 1H, Py-*H*), 7.26 – 7.16 (m, 8H, Ar-*H*), 7.01 – 6.89 (m, 8H, Ar-*H*), 6.72 (dd, 7.5Hz, 2.0 Hz, 1H, Py-*H*), 6.62 (d,10 Hz, 1H, Py-*H* ), 3.88 – 3.83 (four s, 12H, O-C*H_3_*) ppm.

**^13^C{^1^H}-NMR** (CD_2_Cl_2_, 63 MHz) *δ*: 184.6 (d, *J*_Rh-C_ = 69 Hz, Rh-*C*O), 181.4 (d, *J*_Rh-C_ = 76 Hz, Rh-*C*O), 166.6, 161.9, 161.3, 161.2, 160.7, 148.3, 144.0, 140.3, 134.7, 134.4, 134.3, 133.4, 133.3, 133.0, 132.9, 132.2, 127.7, 127.6, 126.7, 115.0, 114.4, 114.3, 56.1, 56.0, 55.9 ppm.

**^1^H-NMR** (CD_2_Cl_2_, 500 MHz) *δ*: 8.76 (s, 1H, Py-*H*), 7.26 – 7.17 (m, 8H, Ar-*H*), 7.01 – 6.90 (m, 8H, Ar-*H*), 6.70 (dd, 7.5Hz, 2.0Hz, 1H, Py-*H*), 6.62 (d,10 Hz, 1H, Py-*H* ), 3.88 – 3.83 (four s, 12H, O-C*H_3_*) ppm.

**EA**: calc. (C_37_H_31_NO_6_ClRh, 724.01 g/mol) C 61.38 H 4.32 N 1.93; found C 60.62 H 4.44 N 1.99

Synthesis of **Py∙Rh**

A mixture of **Pz** (106 mg, 0.2 mmol, 1 equiv.) and di-μ-chloro-tetracarbonyldirhodium(I) (81 mg, 0.21 mmol, 1 equiv.) was added to an oven-dried Schlenk flask and dissolved in dry DCM (20 mL). The reaction mixture was stirred at room temperature for one hour. During this time, the color of the mixture changed from orange-red to blue. All the volatiles were removed under vacuum. 5 mL of diethyl ether (Et_2_O) was added to the residue and obtained solid was collected by filtration, washed with 5 mL of Et_2_O, yielding the compound **Pz∙Rh**. Single crystals suitable for XRD were obtained by diffusion of Et_2_O into concentration DCM solution of **Pz∙Rh**. Yield: 151 mg (0.164 mmol, 82%).

**^1^H-NMR** (CD_2_Cl_2_, 250 MHz) *δ*: 8.40 (s, 2H, Pz-*H*), 7.32 – 7.29 (multiplate with broad signal, 8H, Ar-*H*), 7.07 – 6.98 (m, 8H, Ar-*H*), 3.92 – 3.88 (two s, 12H, O-C*H_3_*) ppm.

**^13^C{^1^H}-NMR** (CD_2_Cl_2_, 63 MHz) *δ*: 163.3, 163.1, 150.9, 137.2, 134.9, 134.8, 131.2, 130.8, 115.5, 114.9, 55.3, 55.2 ppm.

*Because of low solubility of **Pz∙Rh** in CD_2_Cl_2_, ^13^C NMR signal corresponding to Rh-*C*O wasn’t observed.

**^1^H-NMR** (CD_2_Cl_2_, 500 MHz) *δ*: 8.40 (s, 2H, Pz-*H*), 7.31 – 7.30 (multiplate with broad signal, 8H, Ar-*H*), 7.06 – 6.99 (m, 8H, Ar-*H*), 3.92 – 3.89 (two s, 12H, O-C*H_3_*) ppm.

**EA**: calc. (C_38_H_30_N_2_O_8_Cl_2_Rh_2_, 919.38 g/mol) C 49.64 H 3.29 N 3.05; found C 48.29 H 3.21 N 3.06

Synthesis of **Py∙Ir**

A mixture of **Py** (106 mg, 0.2 mmol, 1 equiv.), (IrCp*Cl_2_)_2_ (80 mg, 0.1 mmol, 0.5 equiv.) and sodium acetate (82 mg, 1 mmol, 5 equiv.) was added to an oven-dried Schlenk flask and dissolved in dry DCE (20 mL). The reaction mixture was heated at 70 °C under static vacuum and stirred for 3 days. During this time, the color of the mixture changed from orange-red to violet-pink. The reaction mixture was passed through a pad of celite and washed with 20 mL of DCM. All the volatiles were removed using rotary evaporator and 15 mL of Et_2_O added to the residue. Formed precipitate was collected by filtration, washed with 10 mL of Et_2_O, and dried under vacuum, yielding the compound **Py∙Ir** as dark brown solid. Single crystals suitable for XRD were obtained by diffusion of Et_2_O into concentration DCM solution of **Py∙Ir**. Yield: 91 mg (0.102 mmol, 51%).

**^1^H-NMR** (CD_2_Cl_2_, 250 MHz) *δ*: 8.78 (s, 1H, Py-*H*), 7.22 – 7.08 (m, 7H, Ar-*H*), 6.97 – 6.87 (m, 6H, Ar-*H*), 6.57 – 6.48 (m, 2H, Py-*H*), 6.24 – 6.20 (m, 2H, Ar-*H*), 3.88 – 3.83 (four s, 12H, O-C*H_3_*), 1.42 (s, 15H, Cp-C*H_3_*) ppm.

**^13^C{^1^H}-NMR** (CD_2_Cl_2_, 63 MHz) *δ*: 168.0, 161.3, 160.7, 159.5, 158.7, 158.6, 144.0, 139.8, 136.3, 133.9, 133.4, 133.3, 132.8, 132.4, 132.3, 132.2, 130.2, 129.5, 126.5, 126.1, 123.6, 114.2, 114.1, 108.9, 89.0, 56.0, 55.9, 8.8 ppm.

**^1^H-NMR** (CD_2_Cl_2_, 500 MHz) *δ*: 8.78 (d, 2.0 Hz, 1H, Py-*H*), 7.22 (d, 2.0 Hz, 1H, Ar-*H*) 7.20 (d, 10 Hz, 2H, Ar-*H*), 7.15 (d, 10 Hz, 2H, Ar-*H*), 7.10 (d, 10 Hz, 2H, Ar-*H*), 6.96 (d, 10 Hz, 2H, Ar-*H*), 6.96 (d, 10 Hz, 2H, Ar-*H*), 6.92 (d, 10 Hz, 2H, Ar-*H*), 6.89 (d, 10 Hz, 2H, Ar-*H*), 6.55 (dd, 10 Hz, 2.0 Hz, 1H, Py-*H*), 6.49 (d, 10 Hz, 1H, Py-*H*), 6.24 – 6.21 (mixture of dd and d, 2H, Ar-*H*), 3.87 (s, 3H, O-C*H_3_*), 3.84 (s, 3H, O-C*H_3_*), 3.82 (s, 3H, O-C*H_3_*), 3.77 (s, 3H, O-C*H_3_*), 3.87 (s, 15H, Cp-C*H*_3_) ppm.

**EA**: calc. (C_45_H_45_ClNO_4_Ir, 891.52 g/mol) C 60.63 H 5.09 N 1.57; found C 59.06 H 5.07 N 1.66

**MS(ESI)**: m/z = 856.30 [M−Cl^−^]^+^.

**HRMS(ESI)**: calc (C_45_H_45_NO_4_Ir) m/z = 856.2975; found m/z = 856.2969.

Synthesis of **Pz∙Ir**

A mixture of **Pz** (53 mg, 0.1 mmol, 1 equiv.), (IrCp*Cl_2_)_2_ (80 mg, 0.1 mmol, 1 equiv.) and sodium acetate (82 mg, 1 mmol, 10 equiv.) was added to an oven-dried Schlenk flask and dissolved in dry DCE (20 mL). The reaction mixture was heated at 70 °C under static vacuum and stirred for 3 days. During this time, the color of the mixture changed from orange-red to blue. The reaction mixture was passed through a pad of celite and washed with 20 mL of DCM. All the volatiles were removed using rotary evaporator and 15 mL of Et_2_O added to the residue. Formed precipitate was collected by filtration, washed with 10 mL of Et_2_O, and dried under vacuum, yielding the compound **Pz∙Ir** as dark blue solid. Single crystals suitable for XRD were obtained by diffusion of Et_2_O into concentration DCM solution of **Pz∙Ir**. Yield: 79 mg (0.063 mmol, 63%).

**^1^H-NMR** (CD_2_Cl_2_, 250 MHz) *δ*: 8.04 (s, 2H, Pz-*H*), 7.22 – 6.99 (multiplate with broad signal, 10H, Ar-*H*), 6.61 (d, 10 Hz, 2H, Ar-*H*), 6.26 (dd, 10 Hz, 2.5 Hz, 2H, Ar-*H*), 3.89 (s, 6H, O-C*H_3_*), 3.78 (s, 6H, O-C*H_3_*), 1.44 (s, 30H, Cp-C*H_3_*) ppm.

**^13^C{^1^H}-NMR** (CD_2_Cl_2_, 63 MHz) *δ*: 163.5, 160.8, 160.5, 160.0, 146.8, 132.8, 131.4, 130.4, 129.5, 126.5, 109.7, 89.2, 56.0, 55.5, 9.0 ppm.

**^1^H-NMR** (CD_2_Cl_2_, 500 MHz) *δ*: 8.04 (s, 2H, Pz-*H*), 7.22 – 7.00 (multiplate with broad signal, 10H, Ar-*H*), 6.61 (d, 10 Hz, 2H, Ar-*H*), 6.26 (dd, 10 Hz, 2.5 Hz, 2H, Ar-*H*), 3.89 (s, 6H, O-C*H_3_*), 3.78 (s, 6H, O-C*H_3_*), 1.44 (s, 30H, Cp-C*H_3_*) ppm.

**EA**: calc. (C_54_H_58_N_2_O_4_Cl_2_Ir_2_, 1254.40 g/mol) C 51.71 H 4.66 N 2.23; found C 50.44 H 4.66 N 2.25

**MS(ESI)**: m/z = 1219.33 [M−Cl^−^]^+^.

**HRMS(ESI)**: calc (C_54_H_58_N_2_O_4_ClIr_2_) m/z = 1219.3331; found m/z = 1219.3329.

Chemical oxidation attempts of **Py∙Ir** and **Pz∙Ir**

Following our recently reported two electron oxidation of phenyl spacer based diradicaloid (**2^Ph^**) to its dicationic species,^[44, S1a]^ similar oxidation attempts were performed on **Py∙Ir** and **Pz∙Ir** to isolate the corresponding dicationic complex.

A mixture of **Py∙Ir** (45 mg, 0.05 mmol, 1 equiv.) or **Pz∙Ir** (63 mg, 0.05 mmol, 1 equiv.) and NOSbF_6_ (29 mg, 0.11 mmol, 2.2 equiv.) was placed in a Schlenk flask, and 10 mL of dry DCM was added to it at -78 °C. The reaction mixture was stirred for 1h at that temperature and then allow to warm gradually to room temperature. In the case of **Py∙Ir** the color of the solution changed from violet-pink to reddish-brown, and in case of **Py∙Ir** from deep blue to black. However, neither of the reactions proceeded cleanly and both resulted in a non-isolable mixture of multiple products. Most likely, the cyclometalated carbon center gets oxidised in these cases. In addition, cleavage of Ir-Cl or Ir-Cp* bond may also occour under the reaction conditions. Further investigations into the oxidation of cyclometallated iridium complexes bearing TH based diradicaloid ligand are currently underway in our laboratory.

**3. NMR Spectroscopy**

**Figure S1:** ^1^H-NMR-Spectrum of **Py∙Rh** (CD_2_Cl_2_, 250 MHz).

**Figure S2:** ^13^C{^1^H}-NMR-Spectrum of **Py∙Rh** (CD_2_Cl_2_, 63 MHz).

**Figure S3:** ^1^H-NMR-Spectrum of **Py∙Rh** (CD_2_Cl_2_, 500 MHz).

**Figure S4:** ^1^H-NMR-Spectrum of **Pz∙Rh** (CD_2_Cl_2_, 250 MHz).

**Figure S5:** ^13^C{^1^H}-NMR-Spectrum of **Pz∙Rh** (CD_2_Cl_2_, 63 MHz).

**Figure S6:** ^1^H-NMR-Spectrum of **Pz∙Rh** (CD_2_Cl_2_, 500 MHz).

**Figure S7:** ^1^H-NMR-Spectrum of **Py∙Ir** (CD_2_Cl_2_, 250 MHz).

**Figure S8:** ^13^C{^1^H}-NMR-Spectrum of **Py∙Ir** (CD_2_Cl_2_, 63 MHz).

**Figure S9:** ^1^H-NMR-Spectrum of **Py∙Ir** (CD_2_Cl_2_, 500 MHz).

**Figure S10:** Selected region of ^1^H-NMR-Spectrum of **Py∙Ir** (CD_2_Cl_2_, 500 MHz).

**Figure S11:** Selected region of ^1^H-NMR-Spectrum of **Py∙Ir** (CD_2_Cl_2_, 500 MHz).

**Figure S12:** ^1^H-NMR-Spectrum of **Pz∙Ir** (CD_2_Cl_2_, 250 MHz).

**Figure S13:** ^13^C{^1^H}-NMR-Spectrum of **Pz∙Ir** (CD_2_Cl_2_, 63 MHz).

**Figure S14:** ^1^H-NMR-Spectrum of **Pz∙Ir** (CD_2_Cl_2_, 500 MHz).

**4. Crystal structures and crystallographic data**

Crystal data and structure refinement of **Py∙Rh** (CCDC #2483673)

**Figure S15:** X-Ray solid-state structure of **Py∙Rh** Ellipsoids are all set to 50% probability. All H atoms are omitted for clarity reasons. Selected bond lengths [Å]: C1‒C2 1.351(3); C2‒C3 1.469(3); C3‒C4 1.315(3); C5‒C7 1.358(3); C2‒N1 1.397(2); C6‒N1 1.282(2); Rh1‒N1 2.1533(15).

| **Table S1**: Crystal data and structure refinement for **Py∙Rh**. | |
| --- | --- |
| Identification code | **Py∙Rh** |
| Empirical formula | C_37_H_31_ClNO_6_Rh |
| Formula weight | 723.99 |
| Temperature/K | 100.02 |
| Crystal system | triclinic |
| Space group | P-1 |
| a/Å | 12.6252(10) |
| b/Å | 12.9312(10) |
| c/Å | 13.3503(18) |
| α/° | 113.170(5) |
| β/° | 111.022(5) |
| γ/° | 100.718(4) |
| Volume/Å^3^ | 1729.2(3) |
| Z | 2 |
| ρ_calc_g/cm^3^ | 1.391 |
| μ/mm^‑1^ | 0.616 |
| F(000) | 740.0 |
| Crystal size/mm^3^ | 0.328 × 0.309 × 0.170 |
| Radiation | MoKα (λ = 0.71073) |
| 2Θ range for data collection/° | 3.706 to 49.998 |
| Index ranges | -15 ≤ h ≤ 15, -15 ≤ k ≤ 15, -15 ≤ l ≤ 15 |
| Reflections collected | 24880 |
| Independent reflections | 6082 [R_int_ = 0.0271, R_sigma_ = 0.0236] |
| Data/restraints/parameters | 6082/0/419 |
| Goodness-of-fit on F^2^ | 1.116 |
| Final R indexes [I>=2σ (I)] | R_1_ = 0.0225, wR_2_ = 0.0577 |
| Final R indexes [all data] | R_1_ = 0.0256, wR_2_ = 0.0591 |
| Largest diff. peak/hole / e Å^-3^ | 0.58/-0.43 |

Crystal data and structure refinement of **Pz∙Rh** (CCDC #2483659)

**Figure S16:** X-Ray solid-state structure of **Pz∙Rh**. Ellipsoids are all set to 50% probability. All H atoms are omitted for clarity reasons. Selected bond lengths [Å]: C1‒C2 1.383(3); C2‒C3 1.434(3); C3‒N1’ 1.305(3); C2‒N1 1.422(3); Rh1‒N1 2.1174(18).

| **Table S2**: Crystal data and structure refinement for **Pz∙Rh**. | |
| --- | --- |
| Identification code | **Pz∙Rh** |
| Empirical formula | C_19_H_15_ClNO_4_Rh |
| Formula weight | 459.68 |
| Temperature/K | 100.00(10) |
| Crystal system | triclinic |
| Space group | P-1 |
| a/Å | 7.3459(2) |
| b/Å | 10.4638(3) |
| c/Å | 11.6846(3) |
| α/° | 81.208(2) |
| β/° | 81.468(2) |
| γ/° | 87.521(2) |
| Volume/Å^3^ | 877.58(4) |
| Z | 2 |
| ρ_calc_g/cm^3^ | 1.740 |
| μ/mm^‑1^ | 1.150 |
| F(000) | 460.0 |
| Crystal size/mm^3^ | 0.165 × 0.118 × 0.092 |
| Radiation | Mo Kα (λ = 0.71073) |
| 2Θ range for data collection/° | 3.564 to 64.384 |
| Index ranges | -10 ≤ h ≤ 10, -14 ≤ k ≤ 14, -17 ≤ l ≤ 16 |
| Reflections collected | 14797 |
| Independent reflections | 5058 [R_int_ = 0.0462, R_sigma_ = 0.0537] |
| Data/restraints/parameters | 5058/0/237 |
| Goodness-of-fit on F^2^ | 1.080 |
| Final R indexes [I>=2σ (I)] | R_1_ = 0.0347, wR_2_ = 0.0764 |
| Final R indexes [all data] | R_1_ = 0.0401, wR_2_ = 0.0792 |
| Largest diff. peak/hole / e Å^-3^ | 1.18/-1.15 |

Crystal data and structure refinement of **Py∙Ir** (CCDC #2483675)

**Figure S17:** X-Ray solid-state structure of **Py∙Ir**. Ellipsoids are all set to 50% probability. All H atoms are omitted for clarity reasons. Selected bond lengths [Å]: C1‒C2 1.373(3); C2‒C3 1.447(3); C3‒C4 1.336(3); C5‒C7 1.382(3); C2‒N1 1.415(3); C6‒N1 1.305(3); Ir1‒N1 2.083(2); Ir1‒C30 2.029(3).

| **Table S3**: Crystal data and structure refinement for **Py∙Ir**. | |
| --- | --- |
| Identification code | **Py∙Ir** |
| Empirical formula | C_45_H_45_ClIrNO_4_ |
| Formula weight | 891.47 |
| Temperature/K | 100.02 |
| Crystal system | triclinic |
| Space group | P-1 |
| a/Å | 10.5962(7) |
| b/Å | 11.0309(8) |
| c/Å | 17.2427(12) |
| α/° | 73.132(3) |
| β/° | 84.762(4) |
| γ/° | 77.527(3) |
| Volume/Å^3^ | 1882.3(2) |
| Z | 2 |
| ρ_calc_g/cm^3^ | 1.573 |
| μ/mm^‑1^ | 3.663 |
| F(000) | 896.0 |
| Crystal size/mm^3^ | 0.24 × 0.146 × 0.114 |
| Radiation | MoKα (λ = 0.71073) |
| 2Θ range for data collection/° | 3.936 to 56.752 |
| Index ranges | -14 ≤ h ≤ 14, -14 ≤ k ≤ 9, -23 ≤ l ≤ 22 |
| Reflections collected | 32671 |
| Independent reflections | 9382 [R_int_ = 0.0317, R_sigma_ = 0.0381] |
| Data/restraints/parameters | 9382/93/478 |
| Goodness-of-fit on F^2^ | 1.024 |
| Final R indexes [I>=2σ (I)] | R_1_ = 0.0237, wR_2_ = 0.0473 |
| Final R indexes [all data] | R_1_ = 0.0309, wR_2_ = 0.0493 |
| Largest diff. peak/hole / e Å^-3^ | 0.86/-0.82 |

Crystal data and structure refinement of **Pz∙Ir** (CCDC #2483667)

**Figure S18:** X-Ray solid-state structure of **Pz∙Ir**. Ellipsoids are all set to 50% probability. All H atoms are omitted for clarity reasons. Selected bond lengths [Å] Molecule **A** (left): C1‒C2 1.391(8); C2‒C3 1.412(9); C3‒N1 1.313(7); C2‒N1 1.412(8); Ir1‒N1 2.092(6); Ir1‒C5 2.021(7). Molecule **B** (right): C28‒C29 1.387(7); C29‒C30 1.439(9); C30‒N2 1.321(7); C29‒N2 1.412(7); Ir2‒N2 2.052(6); Ir2‒C36 2.036(6).

| **Table S4**: Crystal data and structure refinement for **Pz∙Ir**. | |
| --- | --- |
| Identification code | **Pz∙Ir** |
| Empirical formula | C_54_H_58_Cl_2_Ir_2_N_2_O_4_ |
| Formula weight | 1254.32 |
| Temperature/K | 150 |
| Crystal system | monoclinic |
| Space group | P2/c |
| a/Å | 25.2247(8) |
| b/Å | 7.5308(2) |
| c/Å | 27.6305(11) |
| α/° | 90 |
| β/° | 115.491(3) |
| γ/° | 90 |
| Volume/Å^3^ | 4737.8(3) |
| Z | 4 |
| ρ_calc_g/cm^3^ | 1.758 |
| μ/mm^‑1^ | 5.773 |
| F(000) | 2464.0 |
| Crystal size/mm^3^ | 0.461 × 0.178 × 0.067 |
| Radiation | Mo Kα (λ = 0.71073) |
| 2Θ range for data collection/° | 2.972 to 54.416 |
| Index ranges | -31 ≤ h ≤ 27, -6 ≤ k ≤ 9, -35 ≤ l ≤ 34 |
| Reflections collected | 53792 |
| Independent reflections | 9912 [R_int_ = 0.0870, R_sigma_ = 0.1103] |
| Data/restraints/parameters | 9912/0/591 |
| Goodness-of-fit on F^2^ | 0.948 |
| Final R indexes [I>=2σ (I)] | R_1_ = 0.0433, wR_2_ = 0.0870 |
| Final R indexes [all data] | R_1_ = 0.0924, wR_2_ = 0.0969 |
| Largest diff. peak/hole / e Å^-3^ | 2.58/-1.97 |

**Figure S19**: Side view of X-Ray solid-state structure of **Pz∙Ir** (molecule A). Angle between the planes contains C1‒C2‒C3‒N1 and C1’‒C2’‒C3’‒N1’ is 41°.

**Figure S20**: Front view of X-Ray solid-state structure of **Pz∙Ir** (molecule A). Angle between the planes contains C2‒C3‒N1’‒C2’ and C2‒N1‒C3’‒C2’ is 27°.

**5. IR Spectroscopy**

**Figure S21:** Selected region of IR spectrum of **Py∙Rh** in DCM at room temperature.

**Figure S22:** Selected region of IR spectrum of **Pz∙Rh** in DCM at room temperature.

**Figure S23:** Comparison of IR spectra of **Py∙Rh**, **Pz∙Rh** and (Rh(CO)_2_Cl)_2_ at room temperature.

**6. UV-Vis-NIR Spectroscopy**

**Figure S24:** UV–vis–NIR spectra of **Py∙Rh** in DCM at room temperature.

**Figure S25:** Linear regression of **Py∙Rh** at 519 nm.

**Figure S26:** UV–vis–NIR spectra of **Pz∙Rh** in DCM at room temperature.

**Figure S27:** Linear regression of **Pz∙Rh** at 522 nm.

**Figure S28:** UV–vis–NIR spectra of **Py∙Ir** in DCM at room temperature.

**Figure S29:** Linear regression of **Py∙Ir** at 524 nm.

**Figure S30:** Linear regression of **Py∙Ir** at 572 nm.

**Figure S31:** UV–vis–NIR spectra of **Pz∙Ir** in DCM at room temperature.

**Figure S32:** Linear regression of **Pz∙Ir** at 622 nm.

**Figure S33:** Normalized UV–vis–NIR spectra of **Py**, **Py∙Rh**, and **Py∙Ir** in DCM at room temperature.

**Figure S34:** Normalized UV–vis–NIR spectra of **Pz**, **Pz∙Rh**, and **Pz∙Ir** in DCM at room temperature.

**Figure S35:** Photograph of DCM solution of **Py**, **Pz**, **Py∙Rh**, **Pz∙Rh**, **Py∙Ir** and **Pz∙Ir** (from left to right) at room temperature.

**7. Cyclic voltammetry and UV-Vis-NIR Spectro-electrochemistry**

**Figure S36:** Cyclic voltammograms of **Py∙Rh** with 0.1 M NBu_4_PF_6_ in DCM at 100 mV/s scan rate.

**Figure S37:** Cyclic voltammograms of **Pz∙Rh** with 0.1 M NBu_4_PF_6_ in DCM at 100 mV/s scan rate.

**Figure S38:** Cyclic voltammograms of **Py∙Ir** with 0.1 M NBu_4_PF_6_ in DCM at 100 mV/s scan rate.

**Figure S39:** Cyclic voltammograms of **Pz∙Ir** with 0.1 M NBu_4_PF_6_ in DCM at 100 mV/s scan rate.

**Figure S40:** Overlay of cyclic voltammograms of **Py∙Ir** and **Pz∙Ir** measured in 0.1 M NBu_4_PF_6_ in DCM at 100 mV/s scan rate.

**Figure S41:** Cyclic voltammograms of **Pz∙Ir** with 0.02 M NBu_4_BArF_24_ in DCM at 100 mV/s scan rate.

**Figure S42:** Changes in the Uv-vis-NIR spectra of **Py∙Ir** in DCM/0.2M Bu_4_NPF_6_ during the 1^st^ oxidation with an Au working electrode.

**Figure S43:** Changes in the Uv-vis-NIR spectra of **Py∙Ir** in DCM/0.2M Bu_4_NPF_6_ during the 2^nd^ oxidation with an Au working electrode.

**Figure S44:** Changes in the Uv-vis-NIR spectra of **Pz∙Ir** in DCM/0.2M Bu_4_NPF_6_ during the 1^st^ oxidation with an Au working electrode.

**Figure S45:** Changes in the Uv-vis-NIR spectra of **Pz∙Ir** in DCM/0.2M Bu_4_NPF_6_ during the 1^st^ oxidation with an Au working electrode.

**Figure S46:** Changes in the selected region of the Uv-vis-NIR spectra (*vs* Wavenumber) of **Py∙Ir** in DCM/0.2M Bu_4_NPF_6_ during the 1^st^ oxidation with an Au working electrode.

**Figure S47:** Changes in the selected region of the Uv-vis-NIR spectra (*vs* Wavenumber) of **Pz∙Ir** in DCM/0.2M Bu_4_NPF_6_ during the 1^st^ oxidation with an Au working electrode.

**Figure S48:** Deconvolution of the selected region of the Uv-vis-NIR spectra (*vs* Wavenumber) of **Pz∙Ir** radical cation.

**Calculation of coupling integral (V) for Pz∙Ir:**

ε_max_ = 2.25×10^4^ M^−1^cm^−1^ (conc: 1.87×10^−5^ M and A_max_ : 0.42 for the oxidised peak) , ῦ_max_ = 9039 cm^−1^, ῦ_1/2_ = 1353 cm^−1^ and R = 5.58 Å (distance between two radical carbon center in the optimised geometry of **Pz∙Ir** radical cation)

*V* = {[2.06 x 10^−2^( ῦ_max_ ῦ_1/2_ε)^1/2^}/R]

V = 1937 cm^−1^

**8. Computational Details**

Theoretical calculations were performed using ORCA 5.0 and 6.0.^[7]^ Geometry optimisations and frequencies were calculated starting from experimentally determined molecular structures with the PBE0^[8]^ functional, def2-SVP and SARC-ZORA-TZVP (for Rh and Ir) basis sets.^[9]^ The optimized geometries were employed for single-point and TD-DFT calculations with the PBE0^[8]^ functional and def2-TZVP^[9]^ basis sets. Implicit solvation was approximated using the SMD^[10]^ method together with the CPCM^[11]^ model. CH_2_Cl_2_ was used as the solvent of choice unless stated otherwise. Population analysis were determined from Löwdin reduced orbital populations. Resolution-of-the-identity (RI) approximations^[12]^ with matching basis-sets (def2/J) ^[13]^ were employed to reduce time of calculation. Grimme’s D3 method was used for dispersion correction. ^[14]^ The geometries were optimised as closed-shell singlet (CSS) and triplet (T) for singlet-triplet gap calculations. Diradical character was calculated following previous reported literature.^[1a]^ Natural orbitals were requested using the keyword UNO, and CUBE-files were generated for frontier orbitals using orca_plot. The visualization software *Chemcraft* was used to plot the orbitals and electron density figures.^[15]^

|  |  |  |
| --- | --- | --- |
| HOMO-2 | HOMO-1 | HOMO |
|  |  |  |
| LUMO | LUMO+1 | LUMO+2 |

**Figure S49:** Frontier Molecular Orbitals of **Py∙Rh**. Isosurface value = 0.05.

**Table S5:** Selected experimental UV-vis-NIR data of **Py∙Rh** and their corresponding TD-DFT calculations.

| **State** | $\boldsymbol{\lambda}{}_{\mathbf{DFT}}\boldsymbol{(}\boldsymbol{\lambda}_{\mathbf{exp}}\boldsymbol{)}$ **/ nm** | $\boldsymbol{f}$ | **Main contributing excitation (%)** | | |
| --- | --- | --- | --- | --- | --- |
| **1** | 538 (519) | 1.70 | HOMO | $\to$ | LUMO (92) |

**Figure S50:** Calculated TD-DFT spectrum with discrete transitions of **Py∙Rh**.

|  |  |  |
| --- | --- | --- |
| HOMO-2 | HOMO-1 | HOMO |
|  |  |  |
| LUMO | LUMO+1 | LUMO+3 |

**Figure S51:** Frontier Molecular Orbitals of **Pz∙Rh**. Isosurface value = 0.05.

**Table S6:** Selected experimental UV-vis-NIR data of **Pz∙Rh** and their corresponding TD-DFT calculations.

| **State** | $\boldsymbol{\lambda}{}_{\mathbf{DFT}}\boldsymbol{(}\boldsymbol{\lambda}_{\mathbf{exp}}\boldsymbol{)}$ **/ nm** | $\boldsymbol{f}$ | **Main contributing excitation (%)** | | |
| --- | --- | --- | --- | --- | --- |
| **1** | 547 (522) | 1.58 | HOMO | $\to$ | LUMO (92) |
| **4** | 436 (356) | 0.17 | HOMO−2 | $\to$ | LUMO (92) |
| **10** | 367 (318) | 0.32 | HOMO | $\to$ | LUMO+3 (56) |

**Figure S52:** Calculated TD-DFT spectrum with discrete transitions of **Pz∙Rh**.

|  |  |  |
| --- | --- | --- |
| HOMO-2 | HOMO-1 | HOMO |
|  |  |  |
| LUMO | LUMO+1 | LUMO+2 |

**Figure S53:** Frontier Molecular Orbitals of **Py∙Ir**. Isosurface value = 0.05.

**Table S7:** Selected experimental UV-vis-NIR data of **Py∙Ir** and their corresponding TD-DFT calculations.

| **State** | $\boldsymbol{\lambda}{}_{\mathbf{DFT}}\boldsymbol{(}\boldsymbol{\lambda}_{\mathbf{exp}}\boldsymbol{)}$ **/ nm** | $\boldsymbol{f}$ | **Main contributing excitation (%)** | | |
| --- | --- | --- | --- | --- | --- |
| **1** | 549 (572) | 1.29 | HOMO | $\to$ | LUMO (84) |
| **2** | 506 (524) | 0.29 | HOMO−1 | $\to$ | LUMO (88) |

**Figure S54:** Calculated TD-DFT spectrum with discrete transitions of **Py∙Ir**.

|  |  |  |
| --- | --- | --- |
| HOMO-2 | HOMO-1 | HOMO |
|  |  |  |
| LUMO | LUMO+1 | LUMO+2 |

**Figure S55:** Frontier Molecular Orbitals of **Pz∙Ir**. Isosurface value = 0.05.

**Table S8:** Selected experimental UV-vis-NIR data of **Pz∙Ir** and their corresponding TD-DFT calculations.

| **State** | $\boldsymbol{\lambda}{}_{\mathbf{DFT}}\boldsymbol{(}\boldsymbol{\lambda}_{\mathbf{exp}}\boldsymbol{)}$ **/ nm** | $\boldsymbol{f}$ | **Main contributing excitation (%)** | | |
| --- | --- | --- | --- | --- | --- |
| **1** | 576 (622) | 1.14 | HOMO | $\to$ | LUMO (88) |
| **2** | 502 (538) | 0.24 | HOMO−1 | $\to$ | LUMO (91) |

**Figure S56:** Calculated TD-DFT spectrum with discrete transitions of **Pz∙Ir**.

|  |  |  |
| --- | --- | --- |
| SOMOα-1 | SOMOα | LUMOα |
|  |  |  |
| LUMOα+1 | SOMOβ−2 | SOMOβ−1 |
|  |  |  |
| SOMOβ | LUMOβ | LUMOβ +1 |

**Figure S57:** Frontier Molecular Orbitals of **Py∙Ir** radical cation. Isosurface value = 0.05.

**Figure S58:** Spin Density Plot of **Py∙Ir** radical cation. Isosurface value = 0.005 (left). Calculated Löwdin spin densities (right).

|  |  |  |
| --- | --- | --- |
| SOMOα-1 | SOMOα | LUMOα |
|  |  |  |
| LUMOα+1 | SOMOβ−2 | SOMOβ−1 |
|  |  |  |
| SOMOβ | LUMOβ | LUMOβ +1 |

**Figure S59:** Frontier Molecular Orbitals of **Pz∙Ir** radical cation. Isosurface value = 0.05.

**Figure S60:** Spin Density Plot of **Pz∙Ir** radical cation. Isosurface value = 0.005 (left). Calculated Löwdin spin densities (right).

**Figure S61:** Energy differences (in kJ/mol) between the complexes optimized as closed-shell singlets (CSS) or triplet (T) states. The quinoid C-C bond lengths for each structure are included.

**Figure S62:** Frontier natural orbitals for the complexes in their CSS and T states. Isovalue = 0.03.

**Table S9:** Frontier Natural Orbital occupations, diradical character, absolute and relative energies and exchange coupling constants for the complexes in their CSS and T optimized geometries.

| **Structure** |  | **N (HONO)** | **N (LUNO)** | **Diradical Character y_0_** | **Final Single Point Energy (Ha)** | **Dispersion Correction (Ha)** | **E_HS (Ha)** | **E_BS (Ha)** | **J (2) (cm^–1^)** | **Singlet-Triplet Relative Energy (Ha)** | **Singlet-Triplet Relative Energy (kJ/mol)** |
| --- | --- | --- | --- | --- | --- | --- | --- | --- | --- | --- | --- |
| **Py∙Rh** | T | N[170]  1.5017 | N[171]  0.4983 | 0.198 | -2503.96876429 | -0.112666 | -2503.847773 | -2503.856098 | -913.45 | 0.01297 | 34.04 |
| **Py∙Rh** | S | 2.0000 | 0.0000 | 0 | -2503.98173090 | -0.112999 | -2503.828859 | -2503.868732 | -4375.10 | 0 | 0 |
| **Pz∙Rh** | T | N[201]  1.7008 | N[202]  0.2992 | 0.060 | -3317.29045709 | -0.134036 | -3317.142686 | -3317.156421 | -1507.01 | 0.01481 | 38.88 |
| **Pz∙Rh** | S | 2.0000 | 0.0000 | 0 | -3317.30526762 | -0.134214 | -3317.127676 | -3317.171053 | -4759.70 | 0 | 0 |
| **Py∙Ir** | T | N[193]  1.6304 | N[194]  0.3696 | 0.098 | -2660.43164923 | -0.147712 | -2660.271285 | -2660.283937 | -1388.24 | 0.01251 | 32.86 |
| **Py∙Ir** | S | 2.0000 | 0.0000 | 0 | -2660.44416375 | -0.148262 | -2660.255252 | -2660.295902 | -4460.40 | 0 | 0 |
| **Pz∙Ir** | T | N[247]  1.8908 | N[248]  0.1092 | 0.007 | -3630.22673705 | -0.212329 | -3629.992769 | -3630.014408 | -2374.42 | 0.01004 | 26.36 |
| **Pz∙Ir** | S | 2.0000 | 0.0000 | 0 | -3630.23677690 | -0.207804 | -3629.983209 | -3630.028973 | -5021.64 | 0 | 0 |

**Table S10:** Optimised molecular structure of **Py∙Rh** in XYZ-coordinates.

| **Atom** | **x** | **y** | **z** |
| --- | --- | --- | --- |
| Rh | -0.92749 | -0.03407 | 5.254579 |
| Cl | -1.29486 | 0.899371 | 3.116039 |
| O | -3.60305 | 1.63601 | 13.36799 |
| O | 4.905021 | 8.3182 | 8.030928 |
| O | 1.828918 | 4.428617 | -0.13798 |
| O | -5.93372 | -1.5976 | 4.756036 |
| N | -0.82655 | 1.915069 | 6.107138 |
| O | -0.26969 | -1.23933 | 7.886301 |
| O | -0.90816 | -2.71981 | 3.996867 |
| C | 0.589089 | 3.885669 | 6.150063 |
| C | 2.37386 | 5.609426 | 6.160115 |
| C | 0.050714 | 2.701992 | 5.556768 |
| H | 0.449839 | 2.371333 | 4.605563 |
| C | 1.599554 | 4.610435 | 4.004474 |
| C | -2.76761 | 1.585773 | 9.296868 |
| C | 2.647394 | 6.877986 | 5.626767 |
| H | 2.178675 | 7.174197 | 4.695622 |
| C | 2.842084 | 4.699639 | 3.368924 |
| H | 3.738283 | 4.801519 | 3.969838 |
| C | -3.34369 | 0.728223 | 7.025373 |
| C | -1.37438 | 2.226776 | 7.360967 |
| C | 2.963391 | 4.632441 | 1.992954 |
| H | 3.945592 | 4.685486 | 1.543246 |
| C | -3.74263 | 1.160992 | 5.758664 |
| H | -3.38018 | 2.109598 | 5.382726 |
| C | -4.0824 | 1.741753 | 9.736244 |
| H | -4.87525 | 1.852073 | 9.005139 |
| C | 0.14742 | 4.127572 | 7.496795 |
| H | 0.512208 | 4.997019 | 8.028521 |
| C | 1.819763 | 4.49283 | 1.205406 |
| C | 1.496421 | 4.672661 | 5.459631 |
| C | -0.78558 | 3.337475 | 8.060025 |
| H | -1.17888 | 3.583478 | 9.036699 |
| C | -3.39654 | 1.628854 | 12.03559 |
| C | 3.854351 | 6.135817 | 8.014215 |
| H | 4.333749 | 5.820524 | 8.93098 |
| C | 2.999145 | 5.265105 | 7.360358 |
| H | 2.839068 | 4.278071 | 7.778537 |
| C | -3.87602 | -0.48399 | 7.492387 |
| H | -3.59109 | -0.84469 | 8.473841 |
| C | -2.07673 | 1.452408 | 11.6164 |
| H | -1.30343 | 1.318087 | 12.36386 |
| C | -1.77322 | 1.433145 | 10.27203 |
| H | -0.74705 | 1.272272 | 9.961484 |
| C | 3.481799 | 7.761728 | 6.273346 |
| H | 3.675526 | 8.746497 | 5.864352 |
| C | -4.61317 | 0.4209 | 4.974497 |
| H | -4.89678 | 0.797092 | 4.001218 |
| C | -5.09999 | -0.79449 | 5.445801 |
| C | 4.096405 | 7.397947 | 7.47435 |
| C | 0.567393 | 4.42395 | 1.818264 |
| H | -0.31637 | 4.336769 | 1.197324 |
| C | -2.44944 | 1.527415 | 7.864875 |
| C | 0.463917 | 4.48222 | 3.190145 |
| H | -0.51906 | 4.459157 | 3.645938 |
| C | -4.40383 | 1.774223 | 11.08369 |
| H | -5.43484 | 1.911586 | 11.38046 |
| C | -4.72623 | -1.23879 | 6.717488 |
| H | -5.11445 | -2.1853 | 7.075499 |
| C | -4.93014 | 1.797791 | 13.83217 |
| H | -5.34604 | 2.757947 | 13.5133 |
| H | -4.8754 | 1.769022 | 14.91844 |
| H | -5.57413 | 0.987763 | 13.47774 |
| C | -0.5309 | -0.76742 | 6.881708 |
| C | 5.555634 | 7.986411 | 9.244402 |
| H | 6.214751 | 7.123325 | 9.114474 |
| H | 4.83082 | 7.775583 | 10.03572 |
| H | 6.147382 | 8.857763 | 9.516328 |
| C | 3.076549 | 4.517885 | -0.8022 |
| H | 2.855823 | 4.453042 | -1.86544 |
| H | 3.736058 | 3.694101 | -0.51537 |
| H | 3.568978 | 5.470762 | -0.58834 |
| C | -0.91148 | -1.69216 | 4.486058 |
| C | -6.30743 | -1.19743 | 3.45053 |
| H | -5.43062 | -1.09264 | 2.804924 |
| H | -6.85925 | -0.25307 | 3.469422 |
| H | -6.95087 | -1.98599 | 3.065938 |

**Table S11:** Optimised molecular structure of **Py∙Rh** as CSS in XYZ-coordinates.

| **Atom** | **x** | **y** | **z** |
| --- | --- | --- | --- |
| Rh | -1.15304 | 0.089126 | 5.014129 |
| Cl | -1.63786 | 1.332362 | 3.075593 |
| O | -3.47891 | 1.650803 | 13.41586 |
| O | 4.762454 | 8.408911 | 8.0619 |
| O | 2.032938 | 4.300255 | -0.11266 |
| O | -5.96852 | -1.61766 | 4.819727 |
| N | -0.88134 | 1.922836 | 6.088152 |
| O | -0.3976 | -1.49356 | 7.417641 |
| O | -1.35196 | -2.36503 | 3.351789 |
| C | 0.585619 | 3.859569 | 6.152505 |
| C | 2.344109 | 5.606824 | 6.174142 |
| C | 0.014578 | 2.693211 | 5.548306 |
| H | 0.38769 | 2.37281 | 4.58341 |
| C | 1.64119 | 4.569505 | 4.014167 |
| C | -2.74521 | 1.574198 | 9.32538 |
| C | 2.5643 | 6.889379 | 5.65188 |
| H | 2.082474 | 7.168438 | 4.722277 |
| C | 2.901143 | 4.697957 | 3.422124 |
| H | 3.767892 | 4.849766 | 4.054614 |
| C | -3.38976 | 0.734473 | 7.0697 |
| C | -1.39419 | 2.211354 | 7.359464 |
| C | 3.075778 | 4.608421 | 2.053433 |
| H | 4.071505 | 4.693196 | 1.640001 |
| C | -3.78778 | 1.155618 | 5.798512 |
| H | -3.42155 | 2.094896 | 5.405071 |
| C | -4.04343 | 1.765543 | 9.795239 |
| H | -4.84749 | 1.895875 | 9.080245 |
| C | 0.150883 | 4.095062 | 7.502202 |
| H | 0.524045 | 4.958713 | 8.037296 |
| C | 1.970792 | 4.399341 | 1.229007 |
| C | 1.494451 | 4.644796 | 5.465878 |
| C | -0.78312 | 3.306221 | 8.064927 |
| H | -1.16803 | 3.543984 | 9.046833 |
| C | -3.30699 | 1.634706 | 12.07752 |
| C | 3.807375 | 6.180644 | 8.026544 |
| H | 4.301932 | 5.877286 | 8.939298 |
| C | 2.988197 | 5.27878 | 7.366356 |
| H | 2.867223 | 4.281989 | 7.774416 |
| C | -3.93012 | -0.46827 | 7.552708 |
| H | -3.64193 | -0.81942 | 8.536446 |
| C | -2.00448 | 1.418771 | 11.62773 |
| H | -1.22223 | 1.260053 | 12.36011 |
| C | -1.73418 | 1.390488 | 10.27727 |
| H | -0.72246 | 1.19782 | 9.939044 |
| C | 3.36274 | 7.801632 | 6.303012 |
| H | 3.516533 | 8.797548 | 5.905682 |
| C | -4.65757 | 0.407162 | 5.022531 |
| H | -4.91519 | 0.766341 | 4.036374 |
| C | -5.14626 | -0.80068 | 5.506641 |
| C | 3.993665 | 7.455457 | 7.498866 |
| C | 0.70209 | 4.289389 | 1.796551 |
| H | -0.15026 | 4.129606 | 1.148469 |
| C | -2.46538 | 1.518214 | 7.884366 |
| C | 0.544004 | 4.378169 | 3.160061 |
| H | -0.45427 | 4.308849 | 3.573925 |
| C | -4.33091 | 1.806554 | 11.14988 |
| H | -5.35067 | 1.972262 | 11.46995 |
| C | -4.78188 | -1.22788 | 6.78645 |
| H | -5.17286 | -2.17079 | 7.14907 |
| C | -4.78087 | 1.851489 | 13.91115 |
| H | -5.18344 | 2.820793 | 13.59859 |
| H | -4.69956 | 1.830151 | 14.99622 |
| H | -5.46062 | 1.057331 | 13.58469 |
| C | -0.69154 | -0.88527 | 6.498524 |
| C | 5.41415 | 8.103929 | 9.271994 |
| H | 6.116891 | 7.273021 | 9.150004 |
| H | 4.696547 | 7.856353 | 10.06115 |
| H | 5.963768 | 8.999605 | 9.554604 |
| C | 3.295305 | 4.39593 | -0.72759 |
| H | 3.120699 | 4.285521 | -1.79603 |
| H | 3.966929 | 3.600517 | -0.38812 |
| H | 3.761541 | 5.368355 | -0.53674 |
| C | -1.27059 | -1.43088 | 3.994615 |
| C | -6.29088 | -1.25993 | 3.494969 |
| H | -5.39095 | -1.16078 | 2.880024 |
| H | -6.85528 | -0.32228 | 3.460661 |
| H | -6.90962 | -2.06616 | 3.105818 |

**Table S12:** Optimised molecular structure of **Py∙Rh** as triplet (T) in XYZ-coordinates.

| **Atom** | **x** | **y** | **z** |
| --- | --- | --- | --- |
| Rh | -1.0562 | -0.06783 | 5.331435 |
| Cl | -1.368 | 0.947207 | 3.237979 |
| O | -2.80491 | 1.566777 | 13.43934 |
| O | 4.573637 | 8.345363 | 8.252848 |
| O | 2.212596 | 4.028902 | -0.20009 |
| O | -6.20013 | -1.2779 | 4.623935 |
| N | -0.82665 | 1.874005 | 6.173164 |
| O | -0.56198 | -1.37357 | 7.959788 |
| O | -1.2903 | -2.70028 | 3.969262 |
| C | 0.443831 | 3.916886 | 6.001457 |
| C | 2.273787 | 5.618049 | 6.09625 |
| C | 0.120422 | 2.620225 | 5.600775 |
| H | 0.645846 | 2.157088 | 4.776755 |
| C | 1.677414 | 4.534586 | 3.901605 |
| C | -2.68536 | 1.565975 | 9.274518 |
| C | 2.722246 | 6.838453 | 5.555856 |
| H | 2.442783 | 7.097488 | 4.541834 |
| C | 2.934933 | 4.757225 | 3.318134 |
| H | 3.768969 | 5.031334 | 3.952485 |
| C | -3.55801 | 0.879487 | 7.008301 |
| C | -1.53677 | 2.361332 | 7.213616 |
| C | 3.153348 | 4.601729 | 1.961775 |
| H | 4.145672 | 4.768829 | 1.564835 |
| C | -3.83715 | 1.301985 | 5.698207 |
| H | -3.34614 | 2.182095 | 5.303763 |
| C | -3.9139 | 1.488012 | 9.945236 |
| H | -4.82798 | 1.454984 | 9.36521 |
| C | -0.28665 | 4.419615 | 7.088108 |
| H | -0.10157 | 5.428463 | 7.437445 |
| C | 2.10504 | 4.210539 | 1.131472 |
| C | 1.468877 | 4.692886 | 5.321662 |
| C | -1.25558 | 3.648587 | 7.683919 |
| H | -1.84287 | 4.036106 | 8.506039 |
| C | -2.83483 | 1.563581 | 12.0909 |
| C | 3.400199 | 6.221867 | 8.172685 |
| H | 3.665399 | 5.955658 | 9.187092 |
| C | 2.63535 | 5.345457 | 7.422003 |
| H | 2.328545 | 4.407732 | 7.870444 |
| C | -4.22391 | -0.28002 | 7.459968 |
| H | -4.00261 | -0.66425 | 8.447941 |
| C | -1.59803 | 1.645352 | 11.44732 |
| H | -0.70117 | 1.690515 | 12.05329 |
| C | -1.52886 | 1.64993 | 10.07506 |
| H | -0.55747 | 1.692649 | 9.596588 |
| C | 3.477146 | 7.720349 | 6.292586 |
| H | 3.801534 | 8.664834 | 5.872348 |
| C | -4.7172 | 0.620984 | 4.880541 |
| H | -4.87386 | 0.97673 | 3.872105 |
| C | -5.34966 | -0.52669 | 5.349363 |
| C | 3.828692 | 7.421291 | 7.610546 |
| C | 0.845267 | 3.975994 | 1.686549 |
| H | 0.038535 | 3.659538 | 1.037209 |
| C | -2.60119 | 1.571761 | 7.831206 |
| C | 0.639327 | 4.134359 | 3.034317 |
| H | -0.35084 | 3.945553 | 3.429369 |
| C | -3.99654 | 1.487767 | 11.32626 |
| H | -4.96975 | 1.441507 | 11.79615 |
| C | -5.09531 | -0.96693 | 6.652361 |
| H | -5.57771 | -1.87459 | 6.99449 |
| C | -4.02975 | 1.47497 | 14.12682 |
| H | -4.6814 | 2.325058 | 13.89877 |
| H | -3.78436 | 1.485722 | 15.18698 |
| H | -4.55445 | 0.544139 | 13.88701 |
| C | -0.75564 | -0.86942 | 6.954916 |
| C | 4.94461 | 8.082515 | 9.584524 |
| H | 5.562091 | 7.180963 | 9.657917 |
| H | 4.067033 | 7.971015 | 10.23016 |
| H | 5.525112 | 8.942133 | 9.913705 |
| C | 3.470038 | 4.236801 | -0.79587 |
| H | 3.3394 | 4.038154 | -1.85786 |
| H | 4.222985 | 3.552335 | -0.39108 |
| H | 3.811766 | 5.268642 | -0.66094 |
| C | -1.19389 | -1.69843 | 4.496413 |
| C | -6.41024 | -0.91712 | 3.277193 |
| H | -5.47114 | -0.91906 | 2.714961 |
| H | -6.87734 | 0.070095 | 3.196961 |
| H | -7.08215 | -1.66713 | 2.864271 |

**Table S13:** Optimised molecular structure of **Pz∙Rh** in XYZ-coordinates.

| **Atom** | **x** | **y** | **z** |
| --- | --- | --- | --- |
| Rh | 0.728544 | 3.650468 | 3.644462 |
| Cl | 0.472537 | 2.310197 | 1.722562 |
| N | 0.991582 | 1.890094 | 4.807513 |
| O | 5.282781 | 5.299185 | 1.581409 |
| O | 5.705807 | 2.916467 | 10.85573 |
| O | 0.844769 | 5.341433 | 6.082189 |
| O | 0.18048 | 6.041237 | 1.97566 |
| C | 0.021431 | 1.030901 | 4.735229 |
| H | -0.72476 | 1.204655 | 3.971682 |
| C | 3.641229 | 3.22156 | 4.770364 |
| C | 3.788139 | 2.619579 | 7.191639 |
| C | 1.942099 | 1.73987 | 5.824731 |
| C | 3.087251 | 2.51282 | 5.91553 |
| C | 3.618684 | 2.661223 | 3.488819 |
| H | 3.19052 | 1.67739 | 3.342937 |
| C | 4.251402 | 4.476675 | 4.925394 |
| H | 4.279062 | 4.936181 | 5.906186 |
| C | 5.18429 | 2.589651 | 7.249549 |
| H | 5.750745 | 2.471194 | 6.333229 |
| C | 5.861459 | 2.674369 | 8.451825 |
| H | 6.941782 | 2.624935 | 8.457946 |
| C | 5.142937 | 2.81678 | 9.640336 |
| C | 4.170218 | 3.314922 | 2.401836 |
| H | 4.14163 | 2.841902 | 1.430194 |
| C | 4.742443 | 4.573923 | 2.573728 |
| C | 3.087027 | 2.774384 | 8.397075 |
| H | 2.005462 | 2.845845 | 8.37838 |
| C | 4.779597 | 5.148548 | 3.848706 |
| H | 5.227545 | 6.128089 | 3.967117 |
| C | 0.388924 | 5.130071 | 2.623229 |
| C | 3.747974 | 2.872903 | 9.600353 |
| H | 3.201557 | 3.005084 | 10.52658 |
| C | 5.241101 | 4.768137 | 0.267674 |
| H | 5.704365 | 5.515482 | -0.37259 |
| H | 4.209973 | 4.597533 | -0.05361 |
| H | 5.803482 | 3.832553 | 0.203858 |
| C | 7.11933 | 2.871422 | 10.94507 |
| H | 7.575228 | 3.695414 | 10.38923 |
| H | 7.506505 | 1.919616 | 10.57119 |
| H | 7.355947 | 2.970368 | 12.00214 |
| C | 0.812048 | 4.689082 | 5.148399 |
| N | 0.76253 | -0.19441 | 6.663307 |
| O | -3.52934 | -3.60282 | 9.889606 |
| O | -3.95159 | -1.22279 | 0.615313 |
| C | 1.732595 | 0.664862 | 6.735666 |
| H | 2.478699 | 0.491264 | 7.499331 |
| C | -1.88704 | -1.52589 | 6.700643 |
| C | -2.03399 | -0.92439 | 4.279336 |
| C | -0.18798 | -0.0442 | 5.646011 |
| C | -1.33308 | -0.81729 | 5.555347 |
| C | -1.86484 | -0.96523 | 7.98204 |
| H | -1.43646 | 0.018498 | 8.127887 |
| C | -2.49701 | -2.78112 | 6.545781 |
| H | -2.52421 | -3.24095 | 5.565127 |
| C | -3.43016 | -0.89482 | 4.221372 |
| H | -3.99668 | -0.77618 | 5.137626 |
| C | -4.10729 | -0.98002 | 3.01913 |
| H | -5.18762 | -0.93083 | 3.012981 |
| C | -3.38874 | -1.1226 | 1.830648 |
| C | -2.41664 | -1.61871 | 9.069005 |
| H | -2.38802 | -1.14579 | 10.04069 |
| C | -2.98897 | -2.87768 | 8.897232 |
| C | -1.33285 | -1.07941 | 3.073898 |
| H | -0.25129 | -1.15081 | 3.092628 |
| C | -3.02558 | -3.45274 | 7.62243 |
| H | -3.47336 | -4.43237 | 7.504151 |
| C | -1.99376 | -1.17839 | 1.870651 |
| H | -1.44731 | -1.31074 | 0.944465 |
| C | -3.49286 | -3.06908 | 11.20238 |
| H | -3.96005 | -3.81443 | 11.84211 |
| H | -2.46295 | -2.89929 | 11.52805 |
| H | -4.05413 | -2.13255 | 11.26166 |
| C | -5.36515 | -1.17836 | 0.525981 |
| H | -5.82068 | -2.00231 | 1.082183 |
| H | -5.75269 | -0.22656 | 0.899487 |
| H | -5.60173 | -1.27784 | -0.53104 |
| Rh | 1.026481 | -1.95475 | 7.825904 |
| Cl | 1.279464 | -0.61472 | 9.747766 |
| O | 0.900086 | -3.64783 | 5.390037 |
| O | 1.578514 | -4.3449 | 9.494339 |
| C | 1.367979 | -3.43409 | 8.846954 |
| C | 0.940542 | -2.99388 | 6.322414 |

**Table S14:** Optimised molecular structure of **Pz∙Rh** as CSS in XYZ-coordinates.

| **Atom** | **x** | **y** | **z** |
| --- | --- | --- | --- |
| Rh | 0.836359 | 3.5392 | 3.471449 |
| Cl | 0.706169 | 2.022498 | 1.685798 |
| N | 1.028352 | 1.87027 | 4.7921 |
| O | 5.21604 | 5.383983 | 1.627446 |
| O | 5.755516 | 2.833701 | 10.86774 |
| O | 0.864001 | 5.454342 | 5.751276 |
| O | 0.36208 | 5.728653 | 1.519077 |
| C | 0.043418 | 1.029288 | 4.718607 |
| H | -0.67752 | 1.202032 | 3.930755 |
| C | 3.669104 | 3.209371 | 4.800265 |
| C | 3.80925 | 2.581551 | 7.213158 |
| C | 1.956943 | 1.72476 | 5.828228 |
| C | 3.10731 | 2.489307 | 5.935015 |
| C | 3.64493 | 2.67095 | 3.51003 |
| H | 3.228408 | 1.6858 | 3.34398 |
| C | 4.259486 | 4.472206 | 4.969807 |
| H | 4.282881 | 4.915847 | 5.95782 |
| C | 5.205742 | 2.578967 | 7.258916 |
| H | 5.763336 | 2.494656 | 6.333721 |
| C | 5.892299 | 2.653403 | 8.456244 |
| H | 6.973354 | 2.627971 | 8.449644 |
| C | 5.183575 | 2.748928 | 9.65389 |
| C | 4.168917 | 3.356391 | 2.428257 |
| H | 4.114231 | 2.904091 | 1.448328 |
| C | 4.715508 | 4.622472 | 2.615077 |
| C | 3.119148 | 2.690463 | 8.430286 |
| H | 2.036502 | 2.729173 | 8.429758 |
| C | 4.762657 | 5.172245 | 3.899966 |
| H | 5.189414 | 6.159726 | 4.025566 |
| C | 0.542006 | 4.898136 | 2.273245 |
| C | 3.788912 | 2.769367 | 9.628494 |
| H | 3.250189 | 2.849076 | 10.56407 |
| C | 5.123283 | 4.89747 | 0.306106 |
| H | 5.538907 | 5.675196 | -0.33114 |
| H | 4.082484 | 4.71185 | 0.023968 |
| H | 5.702572 | 3.977453 | 0.179396 |
| C | 7.162088 | 2.822811 | 10.94383 |
| H | 7.59757 | 3.669025 | 10.40264 |
| H | 7.576074 | 1.888889 | 10.55023 |
| H | 7.40797 | 2.906802 | 12.00039 |
| C | 0.856074 | 4.721025 | 4.878603 |
| N | 0.725485 | -0.17513 | 6.678322 |
| O | -3.46197 | -3.68871 | 9.843365 |
| O | -4.00398 | -1.13767 | 0.604022 |
| C | 1.710375 | 0.665915 | 6.751867 |
| H | 2.431366 | 0.493107 | 7.539662 |
| C | -1.91531 | -1.51403 | 6.670476 |
| C | -2.0561 | -0.88587 | 4.257762 |
| C | -0.20324 | -0.02948 | 5.642326 |
| C | -1.35368 | -0.79388 | 5.53568 |
| C | -1.89105 | -0.97562 | 7.960698 |
| H | -1.47445 | 0.009492 | 8.126725 |
| C | -2.50573 | -2.77684 | 6.500939 |
| H | -2.52922 | -3.22047 | 5.512927 |
| C | -3.45261 | -0.88296 | 4.212608 |
| H | -4.00978 | -0.79852 | 5.138043 |
| C | -4.1397 | -0.95726 | 3.015578 |
| H | -5.22074 | -0.93161 | 3.022647 |
| C | -3.4315 | -1.05295 | 1.817638 |
| C | -2.4149 | -1.6611 | 9.04251 |
| H | -2.36011 | -1.20885 | 10.02246 |
| C | -2.96153 | -2.92717 | 8.855706 |
| C | -1.36653 | -0.99496 | 3.04036 |
| H | -0.2839 | -1.03392 | 3.040424 |
| C | -3.00876 | -3.47692 | 7.570816 |
| H | -3.43548 | -4.46442 | 7.445242 |
| C | -2.03683 | -1.07369 | 1.842436 |
| H | -1.49852 | -1.15353 | 0.906634 |
| C | -3.36959 | -3.20198 | 11.16464 |
| H | -3.78551 | -3.97955 | 11.80189 |
| H | -2.32886 | -3.01644 | 11.4471 |
| H | -3.9488 | -2.28187 | 11.29102 |
| C | -5.41057 | -1.12621 | 0.528516 |
| H | -5.84616 | -1.97228 | 1.069863 |
| H | -5.82403 | -0.19214 | 0.922316 |
| H | -5.65696 | -1.21009 | -0.52794 |
| Rh | 0.917728 | -1.84449 | 7.998343 |
| Cl | 1.048812 | -0.32848 | 9.784503 |
| O | 0.888491 | -3.75865 | 5.717722 |
| O | 1.394048 | -4.03456 | 9.949564 |
| C | 1.213229 | -3.20384 | 9.195834 |
| C | 0.897013 | -3.02573 | 6.590717 |

**Table S15:** Optimised molecular structure of **Pz∙Rh** as triplet (T) in XYZ-coordinates.

| **Atom** | **x** | **y** | **z** |
| --- | --- | --- | --- |
| Rh | 0.762285 | 3.811024 | 3.869699 |
| Cl | 0.32359 | 2.468115 | 2.000037 |
| N | 0.928906 | 2.024841 | 5.014405 |
| O | 5.217426 | 5.11357 | 1.422963 |
| O | 5.527375 | 2.862136 | 10.99575 |
| O | 1.210522 | 5.536296 | 6.256204 |
| O | 0.336419 | 6.18875 | 2.136116 |
| C | -0.11586 | 1.212046 | 4.942428 |
| H | -0.92571 | 1.518223 | 4.295555 |
| C | 3.714763 | 3.136008 | 4.758586 |
| C | 3.792973 | 2.606306 | 7.223755 |
| C | 1.969284 | 1.679942 | 5.81365 |
| C | 3.161832 | 2.499967 | 5.928384 |
| C | 3.530069 | 2.598153 | 3.474409 |
| H | 2.989317 | 1.668277 | 3.353295 |
| C | 4.43505 | 4.34531 | 4.847835 |
| H | 4.563091 | 4.811815 | 5.816652 |
| C | 5.182384 | 2.753479 | 7.351975 |
| H | 5.795357 | 2.764883 | 6.459261 |
| C | 5.796324 | 2.841973 | 8.586718 |
| H | 6.872755 | 2.934956 | 8.635839 |
| C | 5.028146 | 2.785281 | 9.748908 |
| C | 4.025116 | 3.217147 | 2.343075 |
| H | 3.835848 | 2.768441 | 1.378256 |
| C | 4.721068 | 4.41697 | 2.4608 |
| C | 3.044616 | 2.546023 | 8.416981 |
| H | 1.968085 | 2.435893 | 8.370565 |
| C | 4.923551 | 4.972078 | 3.728995 |
| H | 5.448824 | 5.916693 | 3.801726 |
| C | 0.497058 | 5.28785 | 2.808066 |
| C | 3.643327 | 2.632706 | 9.648381 |
| H | 3.057432 | 2.579694 | 10.55725 |
| C | 4.967118 | 4.628085 | 0.122478 |
| H | 5.410994 | 5.350055 | -0.55984 |
| H | 3.892964 | 4.553789 | -0.07289 |
| H | 5.431989 | 3.649405 | -0.03363 |
| C | 6.920606 | 3.002829 | 11.14624 |
| H | 7.28153 | 3.924676 | 10.67856 |
| H | 7.455945 | 2.1475 | 10.72149 |
| H | 7.106146 | 3.046383 | 12.2175 |
| C | 1.042584 | 4.870897 | 5.346541 |
| N | 0.826396 | -0.32979 | 6.454851 |
| O | -3.45865 | -3.42506 | 10.04517 |
| O | -3.7852 | -1.15778 | 0.479903 |
| C | 1.871029 | 0.483279 | 6.527162 |
| H | 2.680764 | 0.177107 | 7.174202 |
| C | -1.95838 | -1.44226 | 6.711527 |
| C | -2.04121 | -0.90802 | 4.247865 |
| C | -0.21428 | 0.0154 | 5.656128 |
| C | -1.40717 | -0.80405 | 5.542132 |
| C | -1.77222 | -0.90678 | 7.996504 |
| H | -1.23118 | 0.022792 | 8.118714 |
| C | -2.67904 | -2.65127 | 6.620962 |
| H | -2.80835 | -3.11595 | 5.651433 |
| C | -3.43107 | -1.05368 | 4.122928 |
| H | -4.04178 | -1.06568 | 5.017192 |
| C | -4.04819 | -1.14007 | 2.889642 |
| H | -5.12483 | -1.23178 | 2.843016 |
| C | -3.28282 | -1.08275 | 1.72561 |
| C | -2.26635 | -1.52758 | 9.127258 |
| H | -2.07599 | -1.08064 | 10.09269 |
| C | -2.96287 | -2.72693 | 9.008124 |
| C | -1.29574 | -0.84687 | 3.052927 |
| H | -0.21902 | -0.73759 | 3.096877 |
| C | -3.16663 | -3.27984 | 7.739149 |
| H | -3.6923 | -4.22414 | 7.665293 |
| C | -1.89763 | -0.93144 | 1.822924 |
| H | -1.31398 | -0.87774 | 0.912651 |
| C | -3.20842 | -2.94109 | 11.34621 |
| H | -3.6527 | -3.66365 | 12.02764 |
| H | -2.1343 | -2.86735 | 11.54197 |
| H | -3.67297 | -1.9624 | 11.50325 |
| C | -5.17876 | -1.29869 | 0.332834 |
| H | -5.53834 | -2.2205 | 0.801607 |
| H | -5.71323 | -0.44336 | 0.758694 |
| H | -5.3671 | -1.34252 | -0.73793 |
| Rh | 0.995163 | -2.11759 | 7.597042 |
| Cl | 1.435319 | -0.77773 | 9.46848 |
| O | 0.544954 | -3.83923 | 5.208304 |
| O | 1.425382 | -4.49807 | 9.325633 |
| C | 1.262934 | -3.59602 | 8.65566 |
| C | 0.713663 | -3.17519 | 6.118818 |

**Table S16:** Optimised molecular structure of **Py∙Ir** in XYZ-coordinates.

| **Atom** | **x** | **y** | **z** |
| --- | --- | --- | --- |
| Ir | 6.371757 | 7.595056 | 13.95229 |
| Cl | 6.903615 | 5.422468 | 13.01173 |
| O | -0.34039 | 12.30308 | 10.40376 |
| O | 10.67765 | 12.70564 | 5.840032 |
| O | 2.251197 | 4.80796 | 15.68964 |
| O | 11.81301 | 4.222304 | 10.33333 |
| N | 6.16189 | 8.273175 | 12.00781 |
| C | 4.408571 | 7.13477 | 13.89986 |
| C | 1.166467 | 10.55902 | 10.01392 |
| H | 0.73819 | 10.47743 | 9.012464 |
| C | 9.75413 | 9.429959 | 7.221822 |
| H | 9.790615 | 8.369545 | 6.961017 |
| C | 10.21615 | 11.72789 | 6.637198 |
| C | 3.930516 | 6.11244 | 14.72198 |
| H | 4.627307 | 5.507974 | 15.30732 |
| C | 7.2355 | 8.202248 | 11.27193 |
| H | 8.021326 | 7.549994 | 11.65627 |
| C | 2.193867 | 9.720996 | 10.42478 |
| H | 2.580953 | 8.965732 | 9.735882 |
| C | 9.185681 | 9.814324 | 8.447071 |
| C | 10.25493 | 10.36318 | 6.319083 |
| H | 10.6721 | 10.0193 | 5.372319 |
| C | 2.214145 | 10.78788 | 12.57521 |
| H | 2.618276 | 10.87856 | 13.58699 |
| C | 3.457767 | 7.879336 | 13.13893 |
| C | 9.674093 | 12.13001 | 7.869001 |
| H | 9.672753 | 13.19381 | 8.116036 |
| C | 5.080708 | 9.074725 | 11.61784 |
| C | 8.869961 | 6.319694 | 9.690906 |
| H | 7.78756 | 6.225566 | 9.578711 |
| C | 0.653893 | 11.53142 | 10.88538 |
| C | 2.740831 | 9.821163 | 11.71342 |
| C | 2.087453 | 7.571797 | 13.28974 |
| H | 1.351049 | 8.133806 | 12.71231 |
| C | 1.629704 | 6.562189 | 14.1257 |
| H | 0.560476 | 6.361699 | 14.195 |
| C | 9.167792 | 11.19086 | 8.751591 |
| H | 8.777076 | 11.52391 | 9.715809 |
| C | 5.353088 | 10.03234 | 10.56948 |
| H | 4.583889 | 10.7689 | 10.33891 |
| C | 7.496099 | 8.991845 | 10.09988 |
| C | 11.00685 | 5.263661 | 10.07224 |
| C | 3.827648 | 8.916518 | 12.1793 |
| C | 2.568084 | 5.81513 | 14.85052 |
| C | 8.675215 | 8.804879 | 9.384602 |
| C | 1.18743 | 11.64349 | 12.17567 |
| H | 0.812591 | 12.39033 | 12.87618 |
| C | 9.470205 | 7.583158 | 9.565647 |
| C | 11.24135 | 12.36205 | 4.595605 |
| H | 11.54548 | 13.30263 | 4.120402 |
| H | 10.51013 | 11.85015 | 3.947943 |
| H | 12.1269 | 11.71602 | 4.717164 |
| C | 6.286095 | 8.355028 | 15.96802 |
| C | 9.614995 | 5.172332 | 9.945198 |
| H | 9.100975 | 4.216089 | 10.0426 |
| C | 10.87749 | 7.645252 | 9.656787 |
| H | 11.37787 | 8.611076 | 9.554856 |
| C | 11.6312 | 6.513615 | 9.912525 |
| H | 12.71765 | 6.569181 | 10.0087 |
| C | 0.896824 | 4.468965 | 15.86074 |
| H | 0.4358 | 4.142866 | 14.91304 |
| H | 0.314312 | 5.313126 | 16.26724 |
| H | 0.868661 | 3.637725 | 16.57641 |
| C | 7.305075 | 7.35122 | 15.8847 |
| C | 6.497227 | 10.00418 | 9.84212 |
| H | 6.642044 | 10.71907 | 9.031303 |
| C | -0.89983 | 13.28731 | 11.23924 |
| H | -1.68257 | 13.78659 | 10.65483 |
| H | -0.14875 | 14.03461 | 11.54668 |
| H | -1.35248 | 12.84428 | 12.14256 |
| C | 6.623899 | 9.411709 | 15.03797 |
| C | 11.23648 | 2.954992 | 10.55101 |
| H | 10.70197 | 2.594685 | 9.656286 |
| H | 10.53755 | 2.972072 | 11.40389 |
| H | 12.06332 | 2.270226 | 10.77533 |
| C | 8.355439 | 7.850487 | 14.99088 |
| C | 7.9452 | 9.103423 | 14.49109 |
| C | 7.398588 | 6.097036 | 16.68577 |
| H | 6.410345 | 5.778849 | 17.04438 |
| H | 8.049142 | 6.239558 | 17.56463 |
| H | 7.818211 | 5.279879 | 16.08275 |
| C | 5.102226 | 8.345638 | 16.87152 |
| H | 4.237745 | 8.834132 | 16.40251 |
| H | 5.340735 | 8.88863 | 17.80049 |
| H | 4.806555 | 7.323442 | 17.13949 |
| C | 9.60884 | 7.111487 | 14.6701 |
| H | 9.38128 | 6.074866 | 14.38003 |
| H | 10.28061 | 7.082448 | 15.54327 |
| H | 10.15074 | 7.581948 | 13.83857 |
| C | 5.886983 | 10.69586 | 14.8533 |
| H | 4.812175 | 10.56445 | 15.03854 |
| H | 6.003849 | 11.07703 | 13.82936 |
| H | 6.258956 | 11.46891 | 15.5464 |
| C | 8.689557 | 9.980893 | 13.54315 |
| H | 9.227783 | 10.76779 | 14.09623 |
| H | 8.007938 | 10.48182 | 12.84115 |
| H | 9.426787 | 9.416219 | 12.95677 |

**Table S17:** Optimised molecular structure of **Py∙Ir** as CSS in XYZ-coordinates.

| **Atom** | **x** | **y** | **z** |
| --- | --- | --- | --- |
| Ir | 6.403514 | 7.64176 | 14.01223 |
| Cl | 6.99546 | 5.511047 | 13.10874 |
| O | -0.24693 | 12.31164 | 10.37988 |
| O | 10.5816 | 12.64918 | 5.761318 |
| O | 2.317638 | 4.705745 | 15.51602 |
| O | 11.79227 | 4.199103 | 10.18013 |
| N | 6.222916 | 8.321374 | 12.06661 |
| C | 4.455972 | 7.135434 | 13.8837 |
| C | 1.293785 | 10.60269 | 10.0082 |
| H | 0.905668 | 10.54305 | 8.998519 |
| C | 9.628533 | 9.404945 | 7.178331 |
| H | 9.613715 | 8.353094 | 6.917259 |
| C | 10.13262 | 11.67568 | 6.581045 |
| C | 3.987733 | 6.073761 | 14.64237 |
| H | 4.684688 | 5.444355 | 15.184 |
| C | 7.285725 | 8.242875 | 11.32644 |
| H | 8.062873 | 7.589592 | 11.70244 |
| C | 2.311728 | 9.772756 | 10.43298 |
| H | 2.732719 | 9.046436 | 9.746861 |
| C | 9.156761 | 9.796931 | 8.430447 |
| C | 10.10301 | 10.32273 | 6.255161 |
| H | 10.44537 | 9.974271 | 5.290198 |
| C | 2.236746 | 10.76881 | 12.59818 |
| H | 2.595119 | 10.82841 | 13.61974 |
| C | 3.515596 | 7.892515 | 13.13762 |
| C | 9.684854 | 12.0871 | 7.836505 |
| H | 9.735841 | 13.14018 | 8.08558 |
| C | 5.143934 | 9.120873 | 11.67413 |
| C | 8.848653 | 6.33575 | 9.762795 |
| H | 7.768176 | 6.260356 | 9.767579 |
| C | 0.741271 | 11.53665 | 10.88218 |
| C | 2.80845 | 9.843149 | 11.73535 |
| C | 2.154481 | 7.565491 | 13.24939 |
| H | 1.426818 | 8.134764 | 12.68454 |
| C | 1.701801 | 6.522184 | 14.0317 |
| H | 0.643327 | 6.305139 | 14.07384 |
| C | 9.205416 | 11.16303 | 8.738264 |
| H | 8.884706 | 11.49516 | 9.71881 |
| C | 5.423808 | 10.08445 | 10.64356 |
| H | 4.66756 | 10.82664 | 10.4285 |
| C | 7.535009 | 9.019886 | 10.14833 |
| C | 10.97897 | 5.251711 | 9.962834 |
| C | 3.891591 | 8.946213 | 12.21123 |
| C | 2.635082 | 5.759153 | 14.72949 |
| C | 8.674001 | 8.803754 | 9.395968 |
| C | 1.218319 | 11.61774 | 12.18637 |
| H | 0.807477 | 12.33171 | 12.8874 |
| C | 9.453432 | 7.574816 | 9.541477 |
| C | 11.05919 | 12.27319 | 4.491945 |
| H | 11.37452 | 13.19217 | 4.001574 |
| H | 10.27545 | 11.79589 | 3.894312 |
| H | 11.91472 | 11.59425 | 4.571736 |
| C | 6.225229 | 8.410579 | 16.02087 |
| C | 9.58995 | 5.187396 | 9.979426 |
| H | 9.071059 | 4.258433 | 10.16971 |
| C | 10.85683 | 7.605917 | 9.491842 |
| H | 11.35748 | 8.549987 | 9.311321 |
| C | 11.60684 | 6.472561 | 9.702896 |
| H | 12.68961 | 6.505727 | 9.689558 |
| C | 0.963977 | 4.340704 | 15.61165 |
| H | 0.553121 | 4.062585 | 14.63514 |
| H | 0.357878 | 5.148205 | 16.03699 |
| H | 0.926428 | 3.477838 | 16.27467 |
| C | 7.236688 | 7.403185 | 15.99176 |
| C | 6.556973 | 10.0499 | 9.914305 |
| H | 6.70804 | 10.76215 | 9.114054 |
| C | -0.8303 | 13.26542 | 11.23224 |
| H | -1.58859 | 13.77747 | 10.6426 |
| H | -0.09272 | 13.99574 | 11.5825 |
| H | -1.30629 | 12.79312 | 12.09858 |
| C | 6.621238 | 9.464581 | 15.12446 |
| C | 11.19406 | 2.966372 | 10.51038 |
| H | 10.57689 | 2.58907 | 9.6884 |
| H | 10.57934 | 3.051726 | 11.41191 |
| H | 12.01205 | 2.272094 | 10.69278 |
| C | 8.334131 | 7.89897 | 15.1647 |
| C | 7.960908 | 9.147658 | 14.64714 |
| C | 7.280214 | 6.145506 | 16.78469 |
| H | 6.286546 | 5.865714 | 17.13499 |
| H | 7.926726 | 6.257577 | 17.6614 |
| H | 7.663458 | 5.322568 | 16.17911 |
| C | 4.988976 | 8.395987 | 16.84528 |
| H | 4.170064 | 8.908545 | 16.34029 |
| H | 5.172262 | 8.896295 | 17.80124 |
| H | 4.657149 | 7.378542 | 17.04888 |
| C | 9.588611 | 7.144222 | 14.90838 |
| H | 9.359882 | 6.143452 | 14.53317 |
| H | 10.1725 | 7.040974 | 15.82795 |
| H | 10.21257 | 7.644392 | 14.16774 |
| C | 5.911603 | 10.75561 | 14.91144 |
| H | 4.839699 | 10.64135 | 15.07442 |
| H | 6.050971 | 11.12137 | 13.89321 |
| H | 6.276578 | 11.52615 | 15.59921 |
| C | 8.749542 | 10.02079 | 13.73672 |
| H | 9.230527 | 10.82643 | 14.30001 |
| H | 8.110073 | 10.48538 | 12.98366 |
| H | 9.528547 | 9.465184 | 13.21452 |

**Table S18:** Optimised molecular structure of **Py∙Ir** as triplet (T) in XYZ-coordinates.

| **Atom** | **x** | **y** | **z** |
| --- | --- | --- | --- |
| Ir | 6.428798 | 7.682362 | 14.02378 |
| Cl | 7.069238 | 5.529438 | 13.21122 |
| O | -0.31807 | 12.00389 | 10.12866 |
| O | 10.55957 | 13.12159 | 6.497548 |
| O | 2.452542 | 4.796004 | 15.85344 |
| O | 11.57148 | 3.815185 | 9.627819 |
| N | 6.191802 | 8.254849 | 12.05424 |
| C | 4.499067 | 7.120853 | 13.97614 |
| C | 0.889384 | 10.01082 | 10.18267 |
| H | 0.236595 | 9.655235 | 9.394506 |
| C | 9.908085 | 9.61625 | 7.36845 |
| H | 10.07653 | 8.620199 | 6.977447 |
| C | 10.14358 | 11.99522 | 7.117376 |
| C | 4.075774 | 6.083425 | 14.78865 |
| H | 4.804702 | 5.44643 | 15.27688 |
| C | 7.300882 | 8.179317 | 11.31084 |
| H | 8.107985 | 7.610216 | 11.75172 |
| C | 1.897408 | 9.216025 | 10.68888 |
| H | 2.040968 | 8.216062 | 10.29442 |
| C | 9.192629 | 9.763888 | 8.565686 |
| C | 10.37689 | 10.70367 | 6.65195 |
| H | 10.90999 | 10.53412 | 5.725928 |
| C | 2.529879 | 10.95358 | 12.19345 |
| H | 3.169808 | 11.32934 | 12.98421 |
| C | 3.51156 | 7.88497 | 13.28087 |
| C | 9.429711 | 12.172 | 8.303689 |
| H | 9.261743 | 13.18093 | 8.661268 |
| C | 5.090523 | 8.891579 | 11.57936 |
| C | 8.755001 | 6.160163 | 9.624724 |
| H | 7.682492 | 6.151683 | 9.771252 |
| C | 0.692643 | 11.29697 | 10.6837 |
| C | 2.739651 | 9.671949 | 11.70572 |
| C | 2.151739 | 7.646662 | 13.5899 |
| H | 1.391015 | 8.255752 | 13.11834 |
| C | 1.754079 | 6.644159 | 14.44451 |
| H | 0.700516 | 6.491909 | 14.63531 |
| C | 8.964508 | 11.08394 | 9.004127 |
| H | 8.427627 | 11.24561 | 9.93151 |
| C | 5.206167 | 9.573035 | 10.3486 |
| H | 4.349684 | 10.11715 | 9.97718 |
| C | 7.454548 | 8.771279 | 10.06666 |
| C | 10.81447 | 4.929264 | 9.542196 |
| C | 3.827212 | 8.823785 | 12.25576 |
| C | 2.727537 | 5.828164 | 15.02695 |
| C | 8.697752 | 8.626522 | 9.309292 |
| C | 1.520013 | 11.76907 | 11.69592 |
| H | 1.390477 | 12.7619 | 12.1052 |
| C | 9.409882 | 7.373676 | 9.35266 |
| C | 11.28613 | 12.98293 | 5.301178 |
| H | 11.53043 | 13.9921 | 4.974662 |
| H | 10.69182 | 12.48741 | 4.525965 |
| H | 12.21257 | 12.42008 | 5.457829 |
| C | 6.250394 | 8.540958 | 15.99984 |
| C | 9.434006 | 4.961433 | 9.720513 |
| H | 8.876501 | 4.063338 | 9.94815 |
| C | 10.80913 | 7.306779 | 9.176277 |
| H | 11.3632 | 8.220991 | 9.002642 |
| C | 11.49348 | 6.118718 | 9.266854 |
| H | 12.57047 | 6.085081 | 9.152389 |
| C | 1.103391 | 4.49993 | 16.11747 |
| H | 0.560348 | 4.252651 | 15.19929 |
| H | 0.59887 | 5.332914 | 16.61897 |
| H | 1.105253 | 3.633792 | 16.77703 |
| C | 7.28747 | 7.559381 | 16.00557 |
| C | 6.357427 | 9.515509 | 9.606841 |
| H | 6.413579 | 10.02289 | 8.6509 |
| C | -0.54988 | 13.3043 | 10.61027 |
| H | -1.39223 | 13.69413 | 10.04172 |
| H | 0.320205 | 13.95144 | 10.45452 |
| H | -0.80592 | 13.29827 | 11.67538 |
| C | 6.606386 | 9.56044 | 15.04977 |
| C | 10.92422 | 2.607819 | 9.954377 |
| H | 10.1882 | 2.329946 | 9.192646 |
| H | 10.42687 | 2.672022 | 10.92752 |
| H | 11.70233 | 1.847842 | 9.995479 |
| C | 8.361438 | 8.045818 | 15.14291 |
| C | 7.949989 | 9.259516 | 14.5732 |
| C | 7.371134 | 6.33868 | 16.85101 |
| H | 6.387297 | 6.047338 | 17.21905 |
| H | 8.0203 | 6.503954 | 17.7172 |
| H | 7.772016 | 5.501799 | 16.27677 |
| C | 5.021476 | 8.536803 | 16.83592 |
| H | 4.168711 | 8.941439 | 16.29006 |
| H | 5.179787 | 9.146486 | 17.73081 |
| H | 4.755572 | 7.528946 | 17.15182 |
| C | 9.632085 | 7.312561 | 14.90536 |
| H | 9.424892 | 6.293904 | 14.56741 |
| H | 10.22278 | 7.255688 | 15.82448 |
| H | 10.23986 | 7.800714 | 14.14365 |
| C | 5.857837 | 10.81972 | 14.78585 |
| H | 4.791597 | 10.6838 | 14.96903 |
| H | 5.978202 | 11.14163 | 13.75003 |
| H | 6.209719 | 11.63222 | 15.43088 |
| C | 8.709286 | 10.11633 | 13.62373 |
| H | 9.165819 | 10.95993 | 14.15078 |
| H | 8.054622 | 10.52824 | 12.85323 |
| H | 9.503868 | 9.564332 | 13.12222 |

**Table S19:** Optimised molecular structure of **Py∙Ir** radical cation in XYZ-coordinates.

| **Atom** | **x** | **y** | **z** |
| --- | --- | --- | --- |
| Ir | 6.427135 | 7.687732 | 13.98584 |
| Cl | 7.085638 | 5.500672 | 13.19633 |
| O | -0.36442 | 12.07363 | 10.27617 |
| O | 10.88174 | 12.91062 | 6.37193 |
| O | 2.411595 | 4.855398 | 15.82483 |
| O | 11.43297 | 3.829944 | 9.836845 |
| N | 6.193925 | 8.234851 | 12.01224 |
| C | 4.504256 | 7.151044 | 13.96168 |
| C | 1.205964 | 10.38138 | 9.936828 |
| H | 0.80981 | 10.28197 | 8.924095 |
| C | 9.940514 | 9.510892 | 7.346327 |
| H | 10.00957 | 8.483278 | 6.983031 |
| C | 10.38416 | 11.86166 | 7.025626 |
| C | 4.060202 | 6.137512 | 14.80654 |
| H | 4.775329 | 5.552343 | 15.38827 |
| C | 7.276346 | 8.138623 | 11.26662 |
| H | 8.069245 | 7.506288 | 11.67043 |
| C | 2.248422 | 9.582459 | 10.37781 |
| H | 2.677017 | 8.837833 | 9.702373 |
| C | 9.25979 | 9.771485 | 8.553866 |
| C | 10.48546 | 10.53157 | 6.582724 |
| H | 10.98024 | 10.28992 | 5.641978 |
| C | 2.151371 | 10.64241 | 12.5372 |
| H | 2.515457 | 10.7429 | 13.56275 |
| C | 3.517678 | 7.875027 | 13.19788 |
| C | 9.722721 | 12.14056 | 8.238245 |
| H | 9.674949 | 13.17682 | 8.578393 |
| C | 5.118915 | 8.987741 | 11.5899 |
| C | 8.668736 | 6.20575 | 9.554013 |
| H | 7.577105 | 6.217115 | 9.524759 |
| C | 0.63252 | 11.33763 | 10.79132 |
| C | 2.742807 | 9.69997 | 11.6881 |
| C | 2.138871 | 7.558488 | 13.39829 |
| H | 1.383366 | 8.092557 | 12.82104 |
| C | 1.726214 | 6.570462 | 14.2593 |
| H | 0.664105 | 6.349255 | 14.36139 |
| C | 9.1657 | 11.11928 | 8.978492 |
| H | 8.686345 | 11.35284 | 9.931808 |
| C | 5.32682 | 9.864715 | 10.48836 |
| H | 4.534135 | 10.55684 | 10.21018 |
| C | 7.490138 | 8.875051 | 10.07545 |
| C | 10.71922 | 4.944122 | 9.692425 |
| C | 3.839671 | 8.84282 | 12.19885 |
| C | 2.702291 | 5.837531 | 14.97448 |
| C | 8.699985 | 8.690496 | 9.340816 |
| C | 1.115485 | 11.46532 | 12.10064 |
| H | 0.69126 | 12.19752 | 12.78802 |
| C | 9.383156 | 7.415997 | 9.411611 |
| C | 11.57363 | 12.70958 | 5.156078 |
| H | 11.89149 | 13.70152 | 4.814584 |
| H | 10.91901 | 12.25473 | 4.395291 |
| H | 12.46066 | 12.07251 | 5.30279 |
| C | 6.295693 | 8.562442 | 15.96044 |
| C | 9.314376 | 4.988361 | 9.690854 |
| H | 8.722345 | 4.078784 | 9.790048 |
| C | 10.7987 | 7.342576 | 9.384542 |
| H | 11.38227 | 8.26129 | 9.297718 |
| C | 11.45194 | 6.137828 | 9.530324 |
| H | 12.54225 | 6.083954 | 9.544296 |
| C | 1.062291 | 4.506339 | 16.06556 |
| H | 0.570269 | 4.156663 | 15.14418 |
| H | 0.498949 | 5.356004 | 16.48269 |
| H | 1.082288 | 3.690271 | 16.79721 |
| C | 7.372559 | 7.618395 | 15.92876 |
| C | 6.4805 | 9.820359 | 9.747923 |
| H | 6.594497 | 10.47055 | 8.879183 |
| C | -0.98697 | 13.04432 | 11.08659 |
| H | -1.75956 | 13.51734 | 10.46844 |
| H | -0.26943 | 13.81282 | 11.41923 |
| H | -1.46125 | 12.58753 | 11.97121 |
| C | 6.566261 | 9.581146 | 14.96902 |
| C | 10.76802 | 2.600216 | 10.04947 |
| H | 10.13228 | 2.336805 | 9.189068 |
| H | 10.15215 | 2.635213 | 10.96238 |
| H | 11.55155 | 1.84281 | 10.16714 |
| C | 8.391177 | 8.128657 | 15.00508 |
| C | 7.903727 | 9.322929 | 14.43552 |
| C | 7.539132 | 6.416692 | 16.79426 |
| H | 6.571547 | 6.056963 | 17.16904 |
| H | 8.175274 | 6.650795 | 17.66367 |
| H | 8.013847 | 5.597258 | 16.23742 |
| C | 5.113077 | 8.527051 | 16.86389 |
| H | 4.222597 | 8.946822 | 16.37738 |
| H | 5.323618 | 9.124904 | 17.76532 |
| H | 4.876882 | 7.503694 | 17.18174 |
| C | 9.687537 | 7.450912 | 14.72587 |
| H | 9.525558 | 6.38702 | 14.49735 |
| H | 10.35424 | 7.513766 | 15.60091 |
| H | 10.20259 | 7.906917 | 13.86999 |
| C | 5.749168 | 10.8029 | 14.71511 |
| H | 4.684376 | 10.61222 | 14.90562 |
| H | 5.848953 | 11.14218 | 13.67475 |
| H | 6.067898 | 11.63041 | 15.37028 |
| C | 8.590005 | 10.18781 | 13.43441 |
| H | 9.079989 | 11.03418 | 13.94234 |
| H | 7.877235 | 10.61053 | 12.71185 |
| H | 9.359889 | 9.637714 | 12.87711 |

**Table S20:** Optimised molecular structure of **Pz∙Ir** in XYZ-coordinates.

| **Atom** | **x** | **y** | **z** |
| --- | --- | --- | --- |
| Ir | -0.81561 | 2.918427 | 3.653238 |
| Cl | -1.69641 | 1.061782 | 2.368737 |
| O | 3.647175 | -0.01322 | 3.244296 |
| N | -2.06457 | 2.298101 | 5.179035 |
| O | 0.882343 | 1.559462 | 12.32196 |
| C | -1.60835 | 2.111187 | 6.489827 |
| C | -0.39789 | 4.941438 | 4.190723 |
| C | 1.569871 | 1.189492 | 3.569934 |
| H | 1.456655 | 1.364975 | 2.500486 |
| C | 0.610357 | 4.492717 | 3.256783 |
| C | 0.589162 | 1.698032 | 4.43467 |
| C | 0.715938 | 1.426598 | 5.826892 |
| C | 0.9385 | 2.610062 | 8.798711 |
| H | 1.396124 | 3.364319 | 8.152912 |
| C | -1.46233 | 4.483217 | 2.168968 |
| C | -3.34842 | 2.290701 | 4.958846 |
| H | -3.66631 | 2.434971 | 3.926428 |
| C | 0.045447 | 1.685749 | 8.245953 |
| C | -0.3029 | 1.771226 | 6.80264 |
| C | 2.67956 | 0.480068 | 4.037182 |
| C | -1.67055 | 4.985937 | 3.469934 |
| C | 1.856806 | 0.714029 | 6.281026 |
| H | 1.959618 | 0.50225 | 7.346971 |
| C | -0.52262 | 0.725174 | 9.096749 |
| H | -1.22217 | -0.00916 | 8.690481 |
| C | 0.657754 | 1.645986 | 10.99784 |
| C | -0.04589 | 4.128325 | 2.03605 |
| C | 1.243731 | 2.603994 | 10.1588 |
| H | 1.933124 | 3.35044 | 10.55407 |
| C | -0.2235 | 0.701995 | 10.45106 |
| H | -0.67621 | -0.03873 | 11.11334 |
| C | 3.5509 | 0.179328 | 1.852287 |
| H | 4.435245 | -0.29527 | 1.409523 |
| H | 3.546295 | 1.251071 | 1.591315 |
| H | 2.642751 | -0.29085 | 1.440069 |
| C | -0.14798 | 5.492808 | 5.554206 |
| H | -0.98864 | 5.285308 | 6.228878 |
| H | -0.0074 | 6.586004 | 5.51814 |
| H | 0.753117 | 5.050216 | 5.999897 |
| C | 2.075664 | 4.457573 | 3.520186 |
| H | 2.288903 | 4.211597 | 4.569 |
| H | 2.511826 | 5.446348 | 3.304451 |
| H | 2.582698 | 3.715716 | 2.890655 |
| C | -2.9545 | 5.463753 | 4.054967 |
| H | -3.82437 | 5.067656 | 3.51422 |
| H | -3.00581 | 6.564146 | 4.025084 |
| H | -3.04304 | 5.162186 | 5.107793 |
| C | 1.743354 | 2.493939 | 12.9274 |
| H | 2.765219 | 2.430453 | 12.51675 |
| H | 1.770608 | 2.247633 | 13.996 |
| H | 1.372094 | 3.525546 | 12.80467 |
| C | 2.826736 | 0.251261 | 5.416911 |
| H | 3.694605 | -0.30527 | 5.776043 |
| C | 0.599159 | 3.656381 | 0.776924 |
| H | 1.573001 | 3.18967 | 0.978332 |
| H | 0.765051 | 4.496833 | 0.082658 |
| H | -0.03185 | 2.916105 | 0.265768 |
| C | -2.478 | 4.301627 | 1.094442 |
| H | -2.43036 | 3.280827 | 0.687568 |
| H | -2.29928 | 5.00836 | 0.268078 |
| H | -3.49606 | 4.465874 | 1.472067 |
| O | -9.59316 | -0.01286 | 9.225867 |
| N | -3.8811 | 2.298106 | 7.291306 |
| O | -6.82733 | 1.559438 | 0.148215 |
| C | -4.33731 | 2.111169 | 5.980509 |
| C | -7.51582 | 1.189802 | 8.900281 |
| H | -7.40269 | 1.365372 | 9.969722 |
| C | -6.53502 | 1.698246 | 8.035583 |
| C | -6.66165 | 1.426644 | 6.643385 |
| C | -6.88492 | 2.609172 | 3.671689 |
| H | -7.34342 | 3.362793 | 4.317611 |
| C | -2.59724 | 2.290781 | 7.511486 |
| H | -2.27934 | 2.435146 | 8.543886 |
| C | -5.99099 | 1.68564 | 4.224354 |
| C | -5.64276 | 1.771262 | 5.667661 |
| C | -8.62546 | 0.48032 | 8.433018 |
| C | -7.80247 | 0.713999 | 6.189236 |
| H | -7.90518 | 0.502066 | 5.123316 |
| C | -5.42165 | 0.726016 | 3.373327 |
| H | -4.72124 | -0.00758 | 3.779446 |
| C | -6.60296 | 1.645896 | 1.472371 |
| C | -7.19007 | 2.603051 | 2.311584 |
| H | -7.88027 | 3.348821 | 1.916442 |
| C | -5.72063 | 0.702817 | 2.018991 |
| H | -5.26701 | -0.03724 | 1.356583 |
| C | -9.49704 | 0.179896 | 10.61786 |
| H | -10.3814 | -0.29461 | 11.06059 |
| H | -9.49245 | 1.251679 | 10.87867 |
| H | -8.58894 | -0.29024 | 11.03024 |
| C | -7.68936 | 2.493076 | -0.45708 |
| H | -8.71119 | 2.428319 | -0.04653 |
| H | -7.71623 | 2.246994 | -1.52574 |
| H | -7.3193 | 3.525077 | -0.33406 |
| C | -8.77249 | 0.251331 | 7.053304 |
| H | -9.64031 | -0.30526 | 6.69415 |
| Ir | -5.13009 | 2.918486 | 8.817238 |
| Cl | -4.24973 | 1.061514 | 10.10159 |
| C | -5.54746 | 4.941646 | 8.279965 |
| C | -6.55583 | 4.492992 | 9.2138 |
| C | -4.4832 | 4.483024 | 10.30172 |
| C | -4.27482 | 4.985864 | 9.000827 |
| C | -5.89971 | 4.128352 | 10.43453 |
| C | -5.7972 | 5.493213 | 6.916527 |
| H | -4.95651 | 5.285662 | 6.241894 |
| H | -5.93762 | 6.586424 | 6.952718 |
| H | -6.69833 | 5.050813 | 6.470736 |
| C | -8.02113 | 4.458139 | 8.950326 |
| H | -8.23437 | 4.212312 | 7.901479 |
| H | -8.45712 | 5.446967 | 9.166161 |
| H | -8.52833 | 3.716326 | 9.579776 |
| C | -2.99078 | 5.463579 | 8.415948 |
| H | -2.12098 | 5.066984 | 8.956453 |
| H | -2.93915 | 6.563945 | 8.4463 |
| H | -2.90237 | 5.162466 | 7.362979 |
| C | -6.54491 | 3.65644 | 11.69359 |
| H | -7.5188 | 3.189852 | 11.49209 |
| H | -6.7108 | 4.496907 | 12.38784 |
| H | -5.91403 | 2.916077 | 12.20478 |
| C | -3.46759 | 4.301143 | 11.37626 |
| H | -3.51537 | 3.280288 | 11.78298 |
| H | -3.64623 | 5.007769 | 12.20273 |
| H | -2.44949 | 4.465302 | 10.99869 |

**Table S21:** Optimised molecular structure of **Pz∙Ir** as CSS in XYZ-coordinates.

| **Atom** | **x** | **y** | **z** |
| --- | --- | --- | --- |
| Ir | -0.75398 | 3.141056 | 3.734557 |
| Cl | -1.70905 | 1.519377 | 2.269763 |
| O | 3.246934 | -0.34889 | 3.085349 |
| N | -2.06594 | 2.461442 | 5.189002 |
| O | 1.039426 | 1.264961 | 12.22305 |
| C | -1.62328 | 2.220826 | 6.498036 |
| C | -0.20636 | 5.091526 | 4.447671 |
| C | 1.384853 | 1.133156 | 3.516163 |
| H | 1.259585 | 1.358949 | 2.46602 |
| C | 0.810678 | 4.614959 | 3.551142 |
| C | 0.503479 | 1.719634 | 4.423199 |
| C | 0.608832 | 1.369028 | 5.787807 |
| C | 1.129966 | 2.301 | 8.70766 |
| H | 1.716481 | 2.913792 | 8.032234 |
| C | -1.16822 | 4.877957 | 2.346982 |
| C | -3.34262 | 2.447545 | 4.958677 |
| H | -3.65026 | 2.615999 | 3.934952 |
| C | 0.018388 | 1.623388 | 8.214198 |
| C | -0.3538 | 1.778501 | 6.791013 |
| C | 2.37799 | 0.252255 | 3.924024 |
| C | -1.41633 | 5.299409 | 3.660944 |
| C | 1.638534 | 0.485471 | 6.179577 |
| H | 1.719441 | 0.200037 | 7.220716 |
| C | -0.70477 | 0.823142 | 9.102168 |
| H | -1.56917 | 0.273095 | 8.750308 |
| C | 0.760965 | 1.419011 | 10.91042 |
| C | 0.209688 | 4.401894 | 2.272009 |
| C | 1.502828 | 2.213886 | 10.03943 |
| H | 2.366419 | 2.765589 | 10.38531 |
| C | -0.34435 | 0.722154 | 10.42943 |
| H | -0.9194 | 0.111564 | 11.11404 |
| C | 3.094817 | -0.11977 | 1.704214 |
| H | 3.850872 | -0.72821 | 1.211036 |
| H | 3.259538 | 0.933612 | 1.452511 |
| H | 2.100812 | -0.41716 | 1.356837 |
| C | -0.00268 | 5.526417 | 5.856351 |
| H | -0.90256 | 5.374 | 6.452546 |
| H | 0.256815 | 6.589311 | 5.90905 |
| H | 0.799685 | 4.959851 | 6.329273 |
| C | 2.240415 | 4.405677 | 3.898035 |
| H | 2.35725 | 4.110962 | 4.940984 |
| H | 2.802485 | 5.330748 | 3.737339 |
| H | 2.68775 | 3.621496 | 3.288541 |
| C | -2.68901 | 5.83703 | 4.208988 |
| H | -3.52528 | 5.663208 | 3.5322 |
| H | -2.61496 | 6.913799 | 4.388992 |
| H | -2.93066 | 5.364898 | 5.162349 |
| C | 2.154067 | 1.944569 | 12.74476 |
| H | 3.082158 | 1.629763 | 12.25536 |
| H | 2.200974 | 1.685877 | 13.80097 |
| H | 2.045998 | 3.030141 | 12.64472 |
| C | 2.515521 | -0.06005 | 5.27826 |
| H | 3.288018 | -0.75324 | 5.586811 |
| C | 0.883107 | 3.960775 | 1.020978 |
| H | 1.820296 | 3.44759 | 1.238612 |
| H | 1.112965 | 4.814967 | 0.375653 |
| H | 0.246017 | 3.272944 | 0.462276 |
| C | -2.11397 | 4.852449 | 1.201493 |
| H | -2.17429 | 3.845233 | 0.781566 |
| H | -1.78022 | 5.534549 | 0.414018 |
| H | -3.11873 | 5.148312 | 1.502225 |
| O | -9.19233 | -0.34924 | 9.385309 |
| N | -3.87972 | 2.461363 | 7.281405 |
| O | -6.98505 | 1.26531 | 0.247359 |
| C | -4.3224 | 2.220754 | 5.972388 |
| C | -7.33038 | 1.132962 | 8.954386 |
| H | -7.20508 | 1.358842 | 10.0045 |
| C | -6.44905 | 1.719428 | 8.047256 |
| C | -6.55434 | 1.368631 | 6.682696 |
| C | -7.07576 | 2.300698 | 3.762946 |
| H | -7.66238 | 2.913305 | 4.438444 |
| C | -2.60305 | 2.4475 | 7.511738 |
| H | -2.29545 | 2.615936 | 8.53548 |
| C | -5.96406 | 1.623182 | 4.256293 |
| C | -5.59183 | 1.778294 | 5.679431 |
| C | -8.32343 | 0.251917 | 8.546601 |
| C | -7.58387 | 0.484824 | 6.291043 |
| H | -7.66465 | 0.199111 | 5.249965 |
| C | -5.24084 | 0.823151 | 3.368179 |
| H | -4.37641 | 0.273054 | 3.719896 |
| C | -6.70662 | 1.419194 | 1.560008 |
| C | -7.44861 | 2.213786 | 2.431158 |
| H | -8.31228 | 2.765436 | 2.085412 |
| C | -5.60123 | 0.722382 | 2.040887 |
| H | -5.02612 | 0.111942 | 1.356194 |
| C | -9.04018 | -0.12015 | 10.76645 |
| H | -9.79612 | -0.72874 | 11.25963 |
| H | -9.20507 | 0.9332 | 11.01819 |
| H | -8.04612 | -0.4174 | 11.11379 |
| C | -8.09972 | 1.944942 | -0.27429 |
| H | -9.02782 | 1.630034 | 0.215036 |
| H | -8.14658 | 1.686363 | -1.33053 |
| H | -7.99168 | 3.030507 | -0.17414 |
| C | -8.46082 | -0.06067 | 7.192416 |
| H | -9.23316 | -0.75409 | 6.883972 |
| Ir | -5.19166 | 3.140896 | 8.735896 |
| Cl | -4.23639 | 1.519295 | 10.20066 |
| C | -5.7387 | 5.091447 | 8.022579 |
| C | -6.75623 | 4.614912 | 8.918498 |
| C | -4.77797 | 4.877661 | 10.12374 |
| C | -4.52914 | 5.299246 | 8.80997 |
| C | -6.15596 | 4.401711 | 10.198 |
| C | -5.94153 | 5.526319 | 6.613765 |
| H | -5.04116 | 5.374277 | 6.018236 |
| H | -6.20138 | 6.589122 | 6.560898 |
| H | -6.74332 | 4.959445 | 6.140241 |
| C | -8.18582 | 4.405834 | 8.570872 |
| H | -8.30226 | 4.111898 | 7.527664 |
| H | -8.74796 | 5.330795 | 8.732008 |
| H | -8.63335 | 3.621194 | 9.179636 |
| C | -3.25603 | 5.836575 | 8.262553 |
| H | -2.42058 | 5.664629 | 8.94082 |
| H | -3.33049 | 6.912909 | 8.0802 |
| H | -3.01272 | 5.362589 | 7.310522 |
| C | -6.83009 | 3.960811 | 11.44874 |
| H | -7.76729 | 3.447802 | 11.23069 |
| H | -7.06005 | 4.815107 | 12.0939 |
| H | -6.19344 | 3.272876 | 12.00781 |
| C | -3.83283 | 4.852159 | 11.26971 |
| H | -3.77253 | 3.844891 | 11.68951 |
| H | -4.16715 | 5.534101 | 12.05707 |
| H | -2.82799 | 5.148277 | 10.96953 |

**Table S22:** Optimised molecular structure of **Pz∙Ir** as triplet (T) in XYZ-coordinates.

| **Atom** | **x** | **y** | **z** |
| --- | --- | --- | --- |
| Ir | -0.84894 | 2.611254 | 3.64926 |
| Cl | -1.58664 | 0.62835 | 2.551036 |
| O | 3.750263 | -0.11754 | 3.570028 |
| N | -2.03614 | 2.074655 | 5.230566 |
| O | 0.757898 | 2.547284 | 12.39622 |
| C | -1.6153 | 1.999328 | 6.520567 |
| C | -0.46773 | 4.708666 | 3.939201 |
| C | 1.595879 | 0.972139 | 3.753822 |
| H | 1.416747 | 0.914275 | 2.688303 |
| C | 0.48011 | 4.19151 | 2.986222 |
| C | 0.621705 | 1.568104 | 4.549622 |
| C | 0.796456 | 1.557956 | 5.964785 |
| C | 0.637033 | 3.24024 | 8.794321 |
| H | 0.874941 | 4.026843 | 8.08655 |
| C | -1.65921 | 4.016425 | 2.067494 |
| C | -3.33646 | 2.014272 | 4.969396 |
| H | -3.60129 | 1.995768 | 3.924194 |
| C | 0.081864 | 2.056952 | 8.32501 |
| C | -0.22952 | 1.906319 | 6.882935 |
| C | 2.772368 | 0.467082 | 4.292629 |
| C | -1.78299 | 4.639531 | 3.31812 |
| C | 2.037389 | 1.093531 | 6.479569 |
| H | 2.200588 | 1.121185 | 7.549986 |
| C | -0.20749 | 1.058443 | 9.259473 |
| H | -0.64683 | 0.125843 | 8.92383 |
| C | 0.574935 | 2.445623 | 11.06054 |
| C | -0.24856 | 3.691217 | 1.867705 |
| C | 0.885128 | 3.446706 | 10.14636 |
| H | 1.314011 | 4.385738 | 10.46957 |
| C | 0.030594 | 1.246163 | 10.60497 |
| H | -0.21176 | 0.47607 | 11.32683 |
| C | 3.529686 | -0.29656 | 2.190736 |
| H | 4.399671 | -0.82912 | 1.810709 |
| H | 3.440029 | 0.6642 | 1.671912 |
| H | 2.627323 | -0.88661 | 2.00444 |
| C | -0.13953 | 5.417827 | 5.206164 |
| H | -0.91151 | 5.262971 | 5.961615 |
| H | -0.04296 | 6.496902 | 5.043965 |
| H | 0.801914 | 5.05481 | 5.61914 |
| C | 1.95708 | 4.208272 | 3.146551 |
| H | 2.248194 | 4.062643 | 4.187006 |
| H | 2.356357 | 5.169602 | 2.808722 |
| H | 2.430819 | 3.419014 | 2.564816 |
| C | -3.03307 | 5.13964 | 3.949433 |
| H | -3.92222 | 4.694149 | 3.503244 |
| H | -3.10614 | 6.226149 | 3.83945 |
| H | -3.05059 | 4.919777 | 5.018557 |
| C | 1.28589 | 3.750078 | 12.89648 |
| H | 2.286994 | 3.946893 | 12.49767 |
| H | 1.34825 | 3.629565 | 13.97643 |
| H | 0.634491 | 4.600648 | 12.66628 |
| C | 3.005808 | 0.567164 | 5.671109 |
| H | 3.937075 | 0.189575 | 6.075234 |
| C | 0.305365 | 3.077354 | 0.631508 |
| H | 1.330407 | 2.739323 | 0.785081 |
| H | 0.308584 | 3.797034 | -0.19332 |
| H | -0.29078 | 2.213414 | 0.332909 |
| C | -2.74248 | 3.680403 | 1.106372 |
| H | -2.6809 | 2.629378 | 0.814118 |
| H | -2.66288 | 4.291856 | 0.20219 |
| H | -3.72955 | 3.843133 | 1.540256 |
| O | -9.69859 | -0.11615 | 8.901329 |
| N | -3.91141 | 2.073418 | 7.240276 |
| O | -6.69843 | 2.547579 | 0.073725 |
| C | -4.33221 | 1.998815 | 5.950164 |
| C | -7.54375 | 0.972542 | 8.717384 |
| H | -7.3648 | 0.915049 | 9.782964 |
| C | -6.56912 | 1.567719 | 7.921509 |
| C | -6.74387 | 1.557312 | 6.506357 |
| C | -6.58215 | 3.239648 | 3.675989 |
| H | -6.81993 | 4.026469 | 4.383557 |
| C | -2.61103 | 2.013278 | 7.501387 |
| H | -2.34608 | 1.993914 | 8.546551 |
| C | -6.02886 | 2.055673 | 4.145776 |
| C | -5.71791 | 1.905131 | 5.588059 |
| C | -8.72037 | 0.467747 | 8.178599 |
| C | -7.98491 | 1.093181 | 5.991551 |
| H | -8.14806 | 1.12063 | 4.921133 |
| C | -5.73981 | 1.056809 | 3.211653 |
| H | -5.30203 | 0.123671 | 3.54784 |
| C | -6.51771 | 2.445326 | 1.409706 |
| C | -6.82809 | 3.446623 | 2.323607 |
| H | -7.25561 | 4.386142 | 2.000008 |
| C | -5.97583 | 1.244992 | 1.865833 |
| H | -5.73346 | 0.474729 | 1.144156 |
| C | -9.47824 | -0.29457 | 10.28074 |
| H | -10.3483 | -0.82691 | 10.66086 |
| H | -9.3886 | 0.666408 | 10.79915 |
| H | -8.57593 | -0.8846 | 10.46741 |
| C | -7.22183 | 3.752109 | -0.42724 |
| H | -8.22397 | 3.950726 | -0.0319 |
| H | -7.28034 | 3.632931 | -1.50755 |
| H | -6.56938 | 4.600933 | -0.1936 |
| C | -8.95362 | 0.567394 | 6.800052 |
| H | -9.88498 | 0.190059 | 6.395923 |
| Ir | -5.09807 | 2.610558 | 8.821558 |
| Cl | -4.36087 | 0.627689 | 9.920121 |
| C | -5.47927 | 4.707889 | 8.530945 |
| C | -6.42623 | 4.191383 | 9.485158 |
| C | -4.28604 | 4.016486 | 10.40186 |
| C | -4.16342 | 4.638855 | 9.15079 |
| C | -5.6965 | 3.691584 | 10.60319 |
| C | -5.8084 | 5.415974 | 7.263606 |
| H | -5.03766 | 5.259054 | 6.507308 |
| H | -5.90338 | 6.495383 | 7.424504 |
| H | -6.75085 | 5.05359 | 6.852351 |
| C | -7.90336 | 4.20819 | 9.32623 |
| H | -8.19541 | 4.062586 | 8.286035 |
| H | -8.30226 | 5.169534 | 9.664472 |
| H | -8.37662 | 3.418963 | 9.908401 |
| C | -2.91378 | 5.138184 | 8.517957 |
| H | -2.02443 | 4.690179 | 8.961262 |
| H | -2.83858 | 6.224325 | 8.630081 |
| H | -2.89909 | 4.920525 | 7.448353 |
| C | -6.24934 | 3.078312 | 11.84016 |
| H | -7.27447 | 2.740118 | 11.68758 |
| H | -6.25191 | 3.798396 | 12.66464 |
| H | -5.65288 | 2.214547 | 12.13865 |
| C | -3.20179 | 3.680976 | 11.36199 |
| H | -3.2638 | 2.630418 | 11.65585 |
| H | -3.27965 | 4.29379 | 12.26541 |
| H | -2.21515 | 3.842357 | 10.92669 |

**Table S23:** Optimised molecular structure of **Pz∙Ir** radical cation in XYZ-coordinates.

| **Atom** | **x** | **y** | **z** |
| --- | --- | --- | --- |
| Ir | -0.87673 | 2.682549 | 3.611402 |
| Cl | -1.65682 | 0.708033 | 2.469549 |
| O | 3.819878 | 0.183732 | 3.43266 |
| N | -2.05796 | 2.1287 | 5.190135 |
| O | 0.537855 | 2.066799 | 12.40106 |
| C | -1.61056 | 2.016348 | 6.487715 |
| C | -0.51625 | 4.764892 | 3.950934 |
| C | 1.626162 | 1.15714 | 3.641948 |
| H | 1.482186 | 1.18462 | 2.562366 |
| C | 0.429195 | 4.295711 | 2.960566 |
| C | 0.613843 | 1.665579 | 4.463378 |
| C | 0.786734 | 1.562612 | 5.892548 |
| C | 0.640845 | 2.981902 | 8.844044 |
| H | 0.932927 | 3.81221 | 8.19576 |
| C | -1.73106 | 4.102566 | 2.073678 |
| C | -3.35499 | 2.093136 | 4.964892 |
| H | -3.6619 | 2.144418 | 3.922268 |
| C | 0.058797 | 1.842345 | 8.282515 |
| C | -0.2467 | 1.823708 | 6.826456 |
| C | 2.819829 | 0.656058 | 4.166986 |
| C | -1.8458 | 4.69867 | 3.345506 |
| C | 2.037149 | 1.079244 | 6.401521 |
| H | 2.181689 | 1.025693 | 7.481607 |
| C | -0.30966 | 0.787375 | 9.13166 |
| H | -0.76956 | -0.1108 | 8.71205 |
| C | 0.430601 | 2.044735 | 11.06266 |
| C | -0.31299 | 3.805532 | 1.840813 |
| C | 0.824659 | 3.093945 | 10.22182 |
| H | 1.264213 | 4.005216 | 10.62768 |
| C | -0.12292 | 0.882863 | 10.50163 |
| H | -0.42079 | 0.070546 | 11.16755 |
| C | 3.695102 | 0.145887 | 2.023189 |
| H | 4.627842 | -0.28666 | 1.64382 |
| H | 3.565049 | 1.159127 | 1.611028 |
| H | 2.845193 | -0.48444 | 1.718403 |
| C | -0.17961 | 5.42692 | 5.244492 |
| H | -0.94668 | 5.228416 | 6.005093 |
| H | -0.10579 | 6.51961 | 5.118138 |
| H | 0.781143 | 5.065098 | 5.634534 |
| C | 1.91089 | 4.354476 | 3.089038 |
| H | 2.234824 | 4.179976 | 4.123617 |
| H | 2.259796 | 5.355028 | 2.786691 |
| H | 2.404978 | 3.614664 | 2.447238 |
| C | -3.09403 | 5.173458 | 4.004416 |
| H | -3.98975 | 4.701018 | 3.579896 |
| H | -3.19285 | 6.263483 | 3.877669 |
| H | -3.07586 | 4.973106 | 5.08426 |
| C | 1.04718 | 3.222854 | 13.02512 |
| H | 2.085341 | 3.427424 | 12.7144 |
| H | 1.027271 | 3.028993 | 14.10438 |
| H | 0.424571 | 4.106402 | 12.80484 |
| C | 3.030126 | 0.641576 | 5.571811 |
| H | 3.973809 | 0.252122 | 5.957705 |
| C | 0.235146 | 3.245271 | 0.573499 |
| H | 1.256627 | 2.866273 | 0.711609 |
| H | 0.262882 | 4.020439 | -0.20959 |
| H | -0.38998 | 2.417777 | 0.211041 |
| C | -2.82664 | 3.790606 | 1.113884 |
| H | -2.74789 | 2.751903 | 0.760641 |
| H | -2.77178 | 4.454101 | 0.235882 |
| H | -3.81523 | 3.914545 | 1.575892 |
| O | -9.76558 | 0.184052 | 9.037921 |
| N | -3.88771 | 2.128693 | 7.28027 |
| O | -6.48358 | 2.06667 | 0.069382 |
| C | -4.33513 | 2.016375 | 5.982689 |
| C | -7.57182 | 1.157346 | 8.828551 |
| H | -7.42783 | 1.184875 | 9.90813 |
| C | -6.55949 | 1.66569 | 8.007081 |
| C | -6.73242 | 1.562677 | 6.577921 |
| C | -6.58669 | 2.981829 | 3.626376 |
| H | -6.87879 | 3.812145 | 4.274639 |
| C | -2.59069 | 2.093093 | 7.50551 |
| H | -2.28376 | 2.144345 | 8.548134 |
| C | -6.00458 | 1.842316 | 4.187929 |
| C | -5.69901 | 1.823731 | 5.643982 |
| C | -8.76552 | 0.65629 | 8.303556 |
| C | -7.98285 | 1.079327 | 6.06899 |
| H | -8.12741 | 1.025731 | 4.988907 |
| C | -5.63612 | 0.787323 | 3.338816 |
| H | -5.17617 | -0.11081 | 3.758457 |
| C | -6.37638 | 2.044621 | 1.407786 |
| C | -6.77048 | 3.093838 | 2.2486 |
| H | -7.21004 | 4.005093 | 1.842702 |
| C | -5.82286 | 0.882768 | 1.96885 |
| H | -5.52495 | 0.070447 | 1.302946 |
| C | -9.64078 | 0.146252 | 10.44739 |
| H | -10.5735 | -0.28625 | 10.82679 |
| H | -9.51068 | 1.159502 | 10.85951 |
| H | -8.79089 | -0.48409 | 10.75218 |
| C | -6.99281 | 3.222747 | -0.55472 |
| H | -8.03099 | 3.427364 | -0.24408 |
| H | -6.97283 | 3.028886 | -1.63398 |
| H | -6.37018 | 4.106266 | -0.33439 |
| C | -8.97584 | 0.641736 | 6.898734 |
| H | -9.91954 | 0.2523 | 6.512863 |
| Ir | -5.06887 | 2.682593 | 8.858983 |
| Cl | -4.28882 | 0.708056 | 10.00081 |
| C | -5.42931 | 4.764938 | 8.519456 |
| C | -6.37469 | 4.295797 | 9.509918 |
| C | -4.21436 | 4.102596 | 10.39661 |
| C | -4.09972 | 4.698681 | 9.124765 |
| C | -5.63241 | 3.805609 | 10.62961 |
| C | -5.76605 | 5.426973 | 7.225924 |
| H | -4.9991 | 5.228368 | 6.465231 |
| H | -5.83973 | 6.519674 | 7.352266 |
| H | -6.72689 | 5.065252 | 6.836011 |
| C | -7.85639 | 4.354593 | 9.381579 |
| H | -8.18042 | 4.180124 | 8.347024 |
| H | -8.20526 | 5.355139 | 9.683987 |
| H | -8.35043 | 3.614765 | 10.0234 |
| C | -2.85154 | 5.173422 | 8.465732 |
| H | -1.95578 | 4.70092 | 8.890125 |
| H | -2.75263 | 6.263437 | 8.592514 |
| H | -2.86984 | 4.973116 | 7.385879 |
| C | -6.18043 | 3.245367 | 11.89698 |
| H | -7.202 | 2.866561 | 11.75902 |
| H | -6.20788 | 4.020495 | 12.68012 |
| H | -5.55539 | 2.417743 | 12.25928 |
| C | -3.1187 | 3.790594 | 11.35631 |
| H | -3.19743 | 2.751873 | 11.7095 |
| H | -3.17349 | 4.454047 | 12.23434 |
| H | -2.13015 | 3.914555 | 10.89422 |

**9. References**

[1]	a) A. Mahata, N. I. Neuman, M. Pech, J. Schmidt, L. I. Domenianni, P. Vöhringer, B. Sarkar, *JACS Au*, **2026**, 6, 1654–1666. b) J. Tönnemann, J. Risse, Z. Grote, R. Scopelliti, K. Severin, *Eur. J. Inorg. Chem*. **2013**, 4558–4562; c) P. N. Bartlett, D. C. Cook, M. W. George, J. Ke, W. Levason, G. Reid, W. Su, W. Zhang, *Phys. Chem. Chem. Phys*. **2010**, *12*, 492– 501.

[2]	G. R. Fulmer, A. J. M. Miller, N. H. Sherden, H. E. Gottlieb, A. Nudelman, B. M. Stoltz, J. E. Bercaw, K. I. Goldberg, *Organometallics* **2010**, *29*, 2176–2179.

[3]	a) M. Krejčik, M. Daněk, F. J. Hartl, *Electroanal. Chem. Interfacial Electrochem*. **1991**, *317*, 179–187; b) J. Klein, A. Stuckmann, S. Sobottka, L. Suntrup, M. van der Meer, P. Hommes, H.-U. Reissig, B. Sarkar, *Chem. Eur. J*. **2017**, *23*, 12314–12325.

[4]	a) A. L. Spek, J. Appl. Crystallogr. **2003**, *36*, 7–13; b) G. M. Sheldrick, *Acta Crystallogr. A* **2008**, *64*, 112–122.

[5]	a) G. M. Sheldrick, SHELXS-97, Program for Crystal Structure Solution and Refinement, University of Göttingen, Göttingen, Germany, 1997; b) G. M. Sheldrick, SHELXL Version 2014/7, Program for Chrystal Structure Solution and Refinement, University of Göttingen, Germany, 2014; c) G. M. Sheldrick, SADABS Ver. 2008/1, SADABS. Program for Empirical Absorption Correction, University of Gottingen, Germany, 2012.

[6]	a) G. M. Sheldrick, *Acta Crystallogr. C* **2015**, *71*, 3–8; b) G. M. Sheldrick, *Acta Crystallogr. A* **2015**, *71*, 3–8; c) SAINT+, Data Integration Engine, Version 8.27b©, Bruker AXS Inc., Madison, Wisconsin, USA, 1997–2012.

[7]	a) F. Neese, *Wiley Interdiscip. Rev.: Comput. Mol. Sci.* **2012**, *2*, 73–78; b) F. Neese, *Wiley Interdiscip. Rev.: Comput. Mol. Sci.* **2022**, *12*, e1606; c) F. Neese, *Wiley Interdiscip. Rev.: Comput. Mol. Sci*. **2025**, *15*, e70019.

[8]	C. Adamo, V. Barone, *J. Chem. Phys.* **1999**, *110*, 6158–6170.

[9]	a) F. Weigend, R. Ahlrichs, *PCCP* **2005**, *7*, 3297–3305; b) J. D. Rolfes, F. Neese, J. Comput. Chem. 2020, 41, 1842–1849.

[10]	A. V. Marenich, C. J. Cramer, D. G. Truhlar, *J. Phys. Chem. B* **2009**, *113*, 6378–6596.

[11]	V. Barone, M. Cossi, *J. Phys. Chem. A* **1998**, *102*, 1995–2001.

[12]	a) T. Petrenko, S. Kossmann, F. Neese, *J. Chem. Phys.* **2011**, *134*, 54116; b) F. Neese, G. Olbrich, *Chem. Phys. Lett.* **2002**, *362*, 170–178; c) R. Izsák, F. Neese, *J. Chem. Phys.* **2011**, *135*, 144105; d) J. L. Whitten, *J. Chem. Phys.* **1973**, *58*, 4496; e) O. Vahtras, J. Almlöf, M.W. Feyereisen, *Chem. Phys. Lett.* **1993**, *213*, 514–518; f) F. Neese, F. Wennmohs, A. Hansen, U. Becker, *Chem. Phys.* **2009**, *356*, 98–109.

[13]	a) K. Eichkorn, O. Treutler, H. Öhm, M. Häser, R. Ahlrichs, *Chem. Phys. Lett.* **1995**, *242*, 652–660; b) K. Eichkorn, F. Weigend, O. Treutler, R. Ahlrichs, *Theor. Chem. Acc.* **1997**, *97*, 119–124; c) F. Weigend, *PCCP* **2006**, *8*, 1057–1065.

[14]	S. Grimme, J. Antony, S. Ehrlich, H. Krieg, *J. Chem. Phys.* **2010**, *132*, 154104.

[15]	"Chemcraft - graphical software for visualization of quantum chemistry computations. https://www.chemcraftprog.com".
